# Supplementary material for: Energetic [1,2,5]oxadiazolo [2,3-a]pyrimidin-8-ium Perchlorates: Synthesis and Characterization
Source: Molecules. 2022 Dec 2;27(23):8443. doi: 10.3390/molecules27238443 (PMC9740163; doi:10.3390/molecules27238443)
Supplement: Supplementary file 1 [file molecules-27-08443-s001.zip › molecules-2050409-supplementary.pdf]

# Supplementary Materials

# Energetic [1,2,5]Oxadiazolo[2,3-*a*]pyrimidin-8-ium Perchlorates: Synthesis and Characterization

**Kirill V. Strizhenko [1], Anastasia D. Smirnova [1,2], Sergei A. Filatov [2], Valery P. Sinditskii [1,2], Adam I. Stash [3], Kyrill Yu. Suponitsky [3], Konstantin A. Monogarov [4], Vitaly G. Kiselev [5], and Aleksei B. Sheremetev [1,*]**

[1] *Zelinsky Institute of Organic Chemistry, Russian Academy of Sciences, 119991 Moscow, Russia*

[2] *Mendeleev University of Chemical Technology, 125047 Moscow, Russia*

[3] *Nesmeyanov Institute of Organoelement Compounds, Russian Academy of Sciences, 119334 Moscow, Russia*
[4] *Semenov Federal Research Center for Chemical Physics, Russian Academy of Sciences, 119991 Moscow, Russia*
[5] *Institute of Chemical Kinetics and Combustion, Russian Academy of Sciences, Siberian Branch, 630090 Novosibirsk, Russia*

**CONTENTS**

Decomposition in non-isothermal conditions 2
Decomposition in isothermal conditions 4
Combustion Study 6
Enthalpies of formation 7
References 9
Copies NMR and HRMS 11

## Decomposition in non-isothermal conditions

Differential scanning calorimetry (DSC) and thermogravimetric analysis (TGA) were performed with a DSC 822e Mettler Toledo in the temperature range of 25–300 °C at different heating rates. The samples of 1–2 mg were analyzed in closed aluminum pans with pierced lids under dynamic nitrogen atmosphere. Kinetic data of the thermal decomposition were calculated using the Kissinger's equation [1], assuming the first order of decomposition reaction:

$$\ln\frac{\phi}{T_{max}^2}=\ln\frac{AR}{E_a}-\frac{E_a}{RT_{\max}}, \qquad \ln\frac{\phi E_a}{T_{max}^2 R}=\ln A-\frac{E_a}{RT_{\max}} \qquad (1)$$

where φ is heating rate, $E_a$ is activation energy, A is preexponential factor, $T_{max}$ is temperature of exotherm maximum.

Examples of the obtained DSC and TGA curves are shown in **Figure S1**–**S3**. The results of the calculation by the Kissinger's method are shown in the **Table S1**.

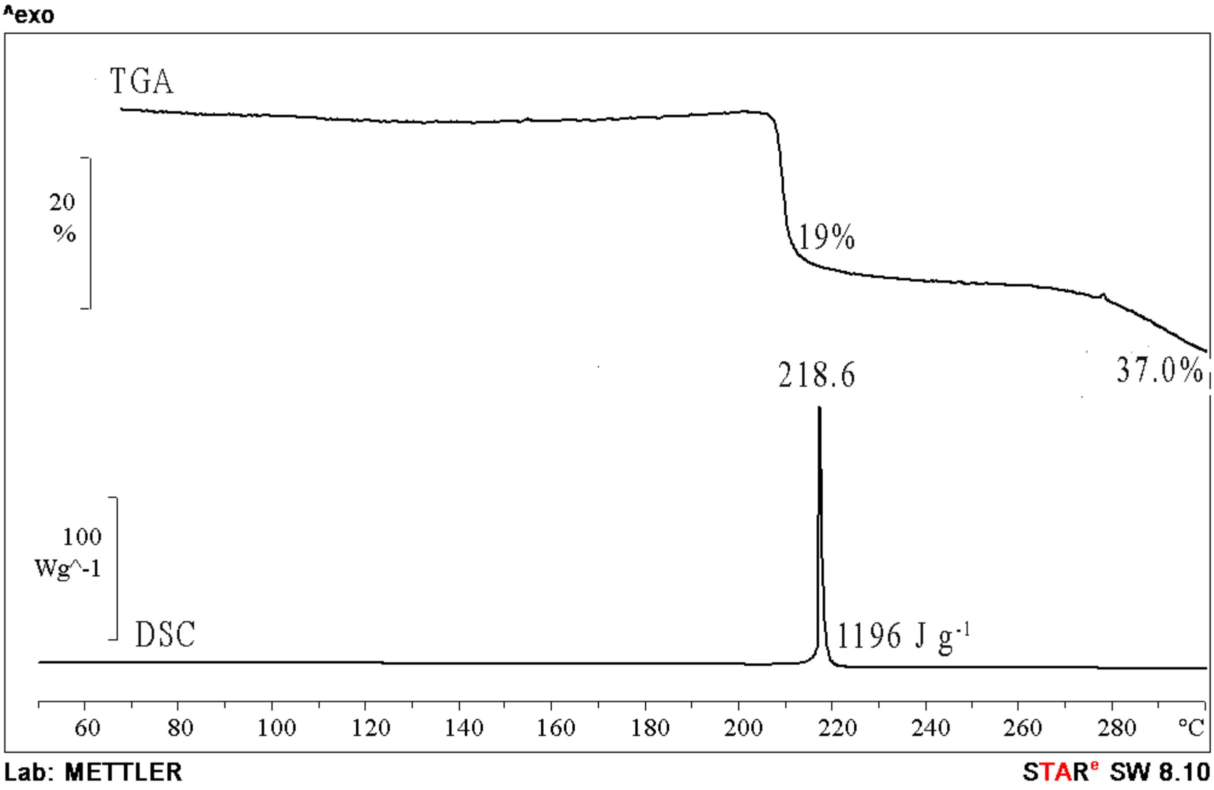


**Figure S1**. TGA and DSC curves of compound **10a** at a heating rate 10 °C min$^{-1}$.

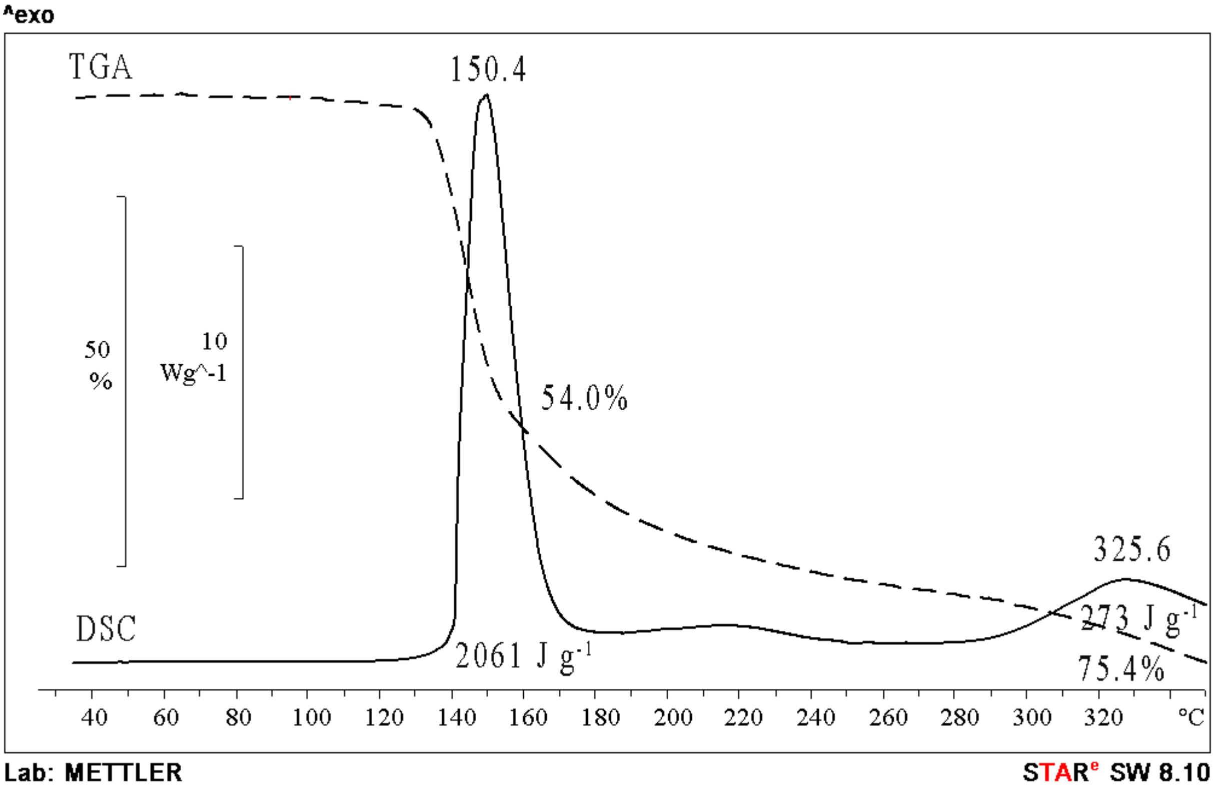


**Figure S2**. TGA and DSC curves of compound **10g** at a heating rate 10 °C min$^{-1}$.

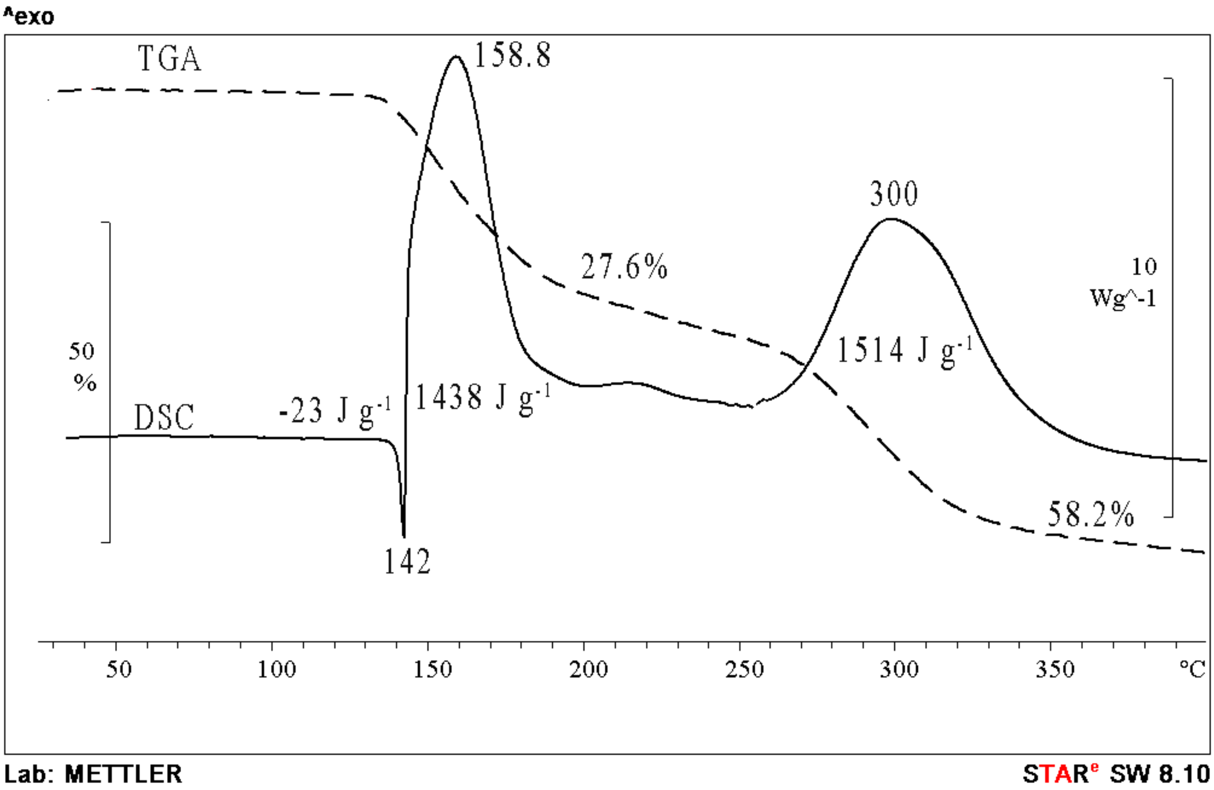


**Figure S3**. TGA and DSC curves of compound **10f** at a heating rate 10 °C min$^{-1}$.

**Table S1**. Results of DSC study for compounds of this study.

| Heating rate, °C $min^{-1}$ | Exotherm temperature (°C) and rate constant ($s^{-1}$) for different compounds | | | | | |
|---|---|---|---|---|---|---|
| | **10a** | | **10g** | | **10f** | |
| | $T_{max}$ | $k{\cdot}10^2$ | $T_{max}$ | $k{\cdot}10^2$ | $T_{max}$ | $k{\cdot}10^2$ |
| 2 | 201 | 0. 27 | 140 | 0. 34 | 142 | 0. 24 |
| 4 | 218 | 0. 51 | 148 | 0. 65 | 152 | 0. 45 |
| 8 | 218 | 1.02 | 153 | 1.27 | 162 | 0.86 |
| 10 | 220 | 1.26 | 151 | 1.61 | 159 | 1.10 |
| 16 | 224 | 2.0 | 161 | 2. 45 | 171 | 1. 66 |

## Decomposition in isothermal conditions

The experiments on the decomposition of perchlorate salts under isothermal conditions were carried out in thin-walled glass manometers of the compensation type (the Bourdon glass gauge). Samples of about 10 mg weight were loaded into a glass manometer with a volume of 10–12 $cm^3$. The ampules were vacuumed to 0.1 Torr, sealed, and put in a thermostat with the Wood's alloy. Pressure of gases evolved in the decomposition experiments (the accuracy of pressure measurements was ±1 mm Hg) was converted to the gas volume (V) at normal conditions. The description of the experimental dependence of gas release on time V(t) by a suitable model allows one to obtain the rate constants.

For compound **10a**, the decomposition under isothermal conditions (**Figure S4**) was carried out in Bourdon gauges at the load density (ratio of sample mass to gauge volume, *m/V*) ~ 1 mg $cm^{-3}$ in the temperature interval of 160–180°C. The decomposition of **10a** produces 60–95 $cm^3$ $g^{-1}$ or 0.7–1.1 moles of gases per one mole of the initial substance before the process stops.

The decomposition of compound **10g** under isothermal conditions (**Figure S5**) was carried out in glass Bourdon gauges (*m/V* ~ 1 mg $cm^{-3}$) in the temperature interval of 80-110°C. The decomposition reaction of compound **10g** also proceeds with acceleration in time and produces about 120 $cm^3$ $g^{-1}$ or 1.58 mol/mol before the process stops.

The decomposition of compound **10f** under isothermal conditions (**Figure S5**) was carried out in glass Bourdon gauges (*m/V* ~ 1 mg $cm^{-3}$) in the temperature interval of 110–130°C before melting. The decomposition reaction of compound **10f** proceeds with acceleration in time and produces about 120 $cm^3$ $g^{-1}$ or 1.66 mol/mol before the process stops.

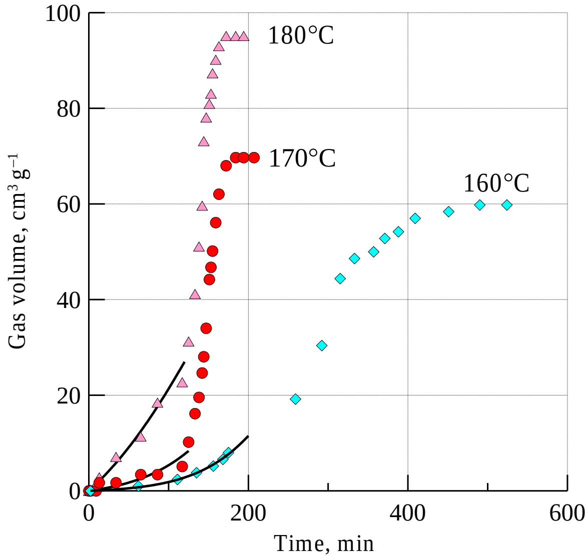


**Figure S4**. Gas release curves of compound **10a** at different temperatures. Points are experiment, lines are fittings

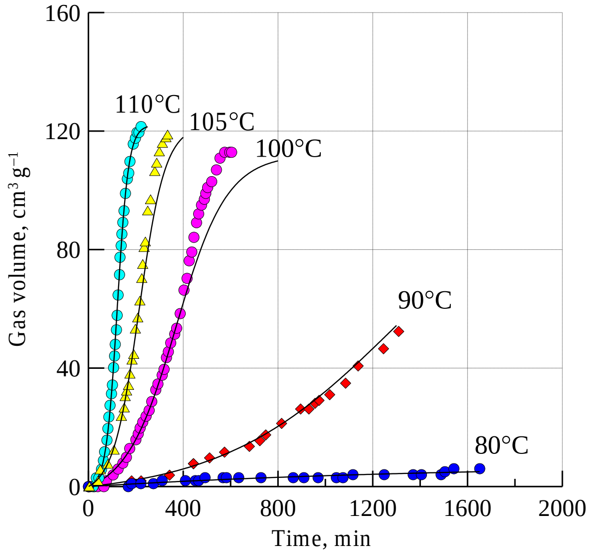


**Figure S5**. Gas release curves of compound **10f** at different temperatures. Points are experiment, lines are fittings

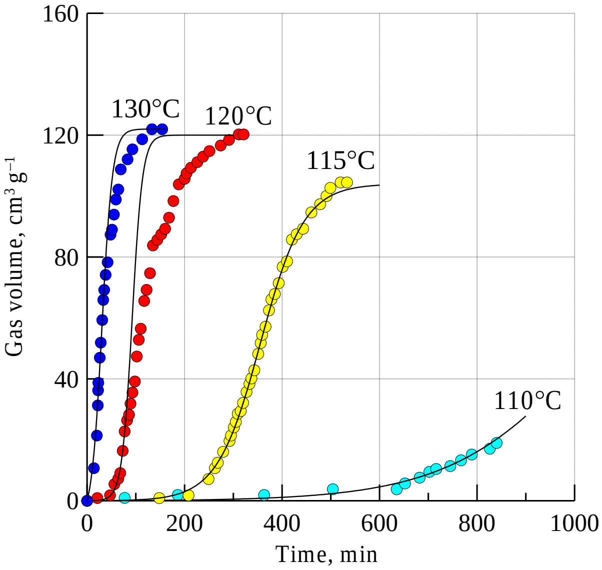


**Figure S6**. Gas release curves of compound **10f**at different temperatures. Points are experiment, lines are fittings

In the view of the complex nature of the decomposition process, the rate constants were calculated using the model of first-order reaction with self-acceleration

$$V = V_{\propto} \cdot k_1(\exp((k_1+k_2)\cdot t)-1)/(k_2 + k_1 \cdot \exp((k_1+k_2)\cdot t)) \qquad (2)$$

where $V_{\propto}$ is maximum volume of gas evolved per gram of compound, $k_1$ is rate constant of non-catalytic stage, $k_2$ is pseudo first-order rate constant of catalytic stage, and $t$ is time.  The results obtained are shown in **Figure S7**.

Comparison of the decomposition rates for compounds of this study depending on the Hammett constants characterizing the inductive effect of substituents at the 1,2,5-oxadiazole ring is shown in **Figure S8**.

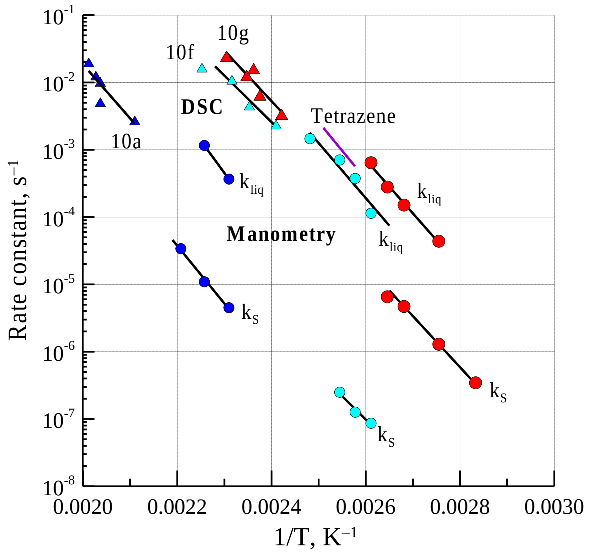


**Figure S7**. Comparison of decomposition kinetic data for compounds **10a**, **10f**, **10g** and tetrazene obtained in non isothermal (DSC, triangles) and isothermal (Manometry, points) conditions.

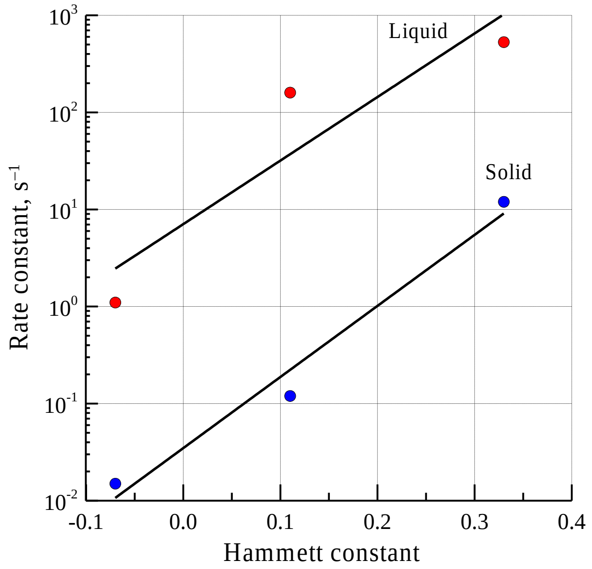


**Figure S8**. Effect of Hammett constants of substituent constants at the 1,2,5-oxadiazole ring on the decomposition rate in liquid and solid state. Temperature is 150°C

## Combustion Study

Burning rates ($r_b$) of perchlorate salts were measured in a window constant-pressure bomb of 1.5-liter volume in the pressure range of 0.1–10 MPa. The bomb was pressurized with nitrogen gas. Samples to test were prepared as pressed cylinders of pure substances of 4–5 mm height confined in transparent acrylic tubes of 4 mm i.d. and 6 mm o.d. Prior to pressing, the material was carefully milled in order to produce samples with a minimum possible pore size, thus minimizing the possibility of flame propagation between particles. The combustion process was recorded with a high-speed video camera. The burning rate was determined by measuring the position of the flame front over the time.

Since the combustion of perchlorates of this study in tubes switched to the convective regime at high pressures, compound **10f** was studied in charges in the form of thin (~1 mm thick) plates pressed to a high density. The use of such charges makes it possible to avoid the penetration of hot gases into pores and to prevent the transition of layer-by-layer combustion to convective.

## Enthalpies of formation

The Born-Haber (BH) thermodynamic cycle (Figure. S9) along with the volume-based thermodynamics (VBT) and quantum chemical calculations were employed to estimate the standard (solid-state) enthalpies of formation $\Delta_f H^0_{solid}$ of the energetic salts studied. More specifically, in the framework of the BH cycle, the enthalpies of formation in the crystalline state read as:

$$\Delta_f H^0_{solid} = -\Delta H_{lat} + \left(\Delta_f H^0_{gas}(An) + \Delta_f H^0_{gas}(Cat)\right), \qquad (3)$$

where $\left(\Delta_f H^0_{gas}(An) + \Delta_f H^0_{gas}(Cat)\right)$ is a sum of the gas phase enthalpies of formation of the ions comprising the salts **10a**, **10f**, and **10g** and $\Delta H_{lat}$ is a lattice enthalpy. The latter value was estimated using the VBT approach via the empirical formula proposed by Jenkins et al. [2,3]:

$$\Delta H_{lat} = 2\left({}^{\alpha}/_{\sqrt[3]{V}} + \beta\right), \qquad (4)$$

where $V$ is the molecular volume of the lattice, which is equal to the sum of the volumes of the perchlorate anion and heterocyclic cations calculated at the B3LYP-D3BJ/def2-TZVPP level, and α and β are empirical parameters.

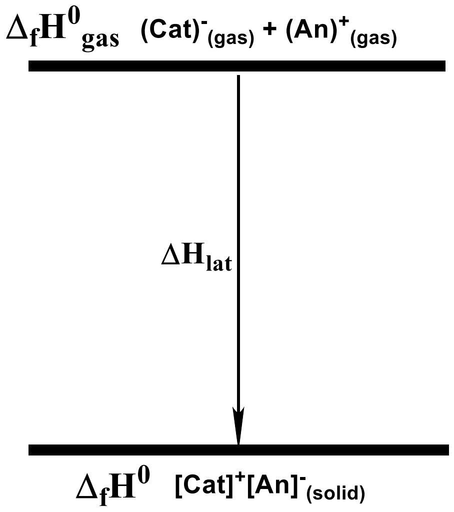


**Figure S9**. The Born-Haber thermodynamic cycle employed for the estimation of the formation enthalpy of the crystalline salts **10a**, **10f**, and **10g**.

The breakdown of the enthalpy components is given in Table S2.

**Table S2**. The Thermochemical Properties of compounds **10a**, **10f**, and **10g**.

| Compound | *V(an)+V(cat)*, [a] mL mol | $\Delta H_f^0$(gas), [b] kJ mol$^{-1}$ | $\Delta H_{lat}$, [c] kJ mol$^{-1}$ | $\Delta_f H^0_{solid}$,, [d] kJ mol$^{-1}$ |
|---|---|---|---|---|
| **10a** | 174.2 | 610.0 | 462.9 | 147.1 |
| **10f** | 203.0 | 998.1 | 445.3 | 552.8 |
| **10g** | 170.1 | 1023.0 | 465.8 | 557.2 |

[a] The individual ion volumes were taken as those inside the 0.001 a.u. contour of the B3LYP-D3BJ/def2-TZVPP electron density. A Gaussian 09 keyword "volume=tight" (i.e., 100 points per cubic Bohr) was used in the Monte Carlo integration to obtain the desired level of accuracy, a reproducible calculated volume to better than 1%. [b] The sum of the gas phase enthalpies of formation of the ions comprising the salts **10a**, **10f**, and **10g** calculated at the W1-F12 level of theory using the atomization energy approach [4]. [c] Calculated using volume-based thermodynamics [2], Eq. (4). [d] Calculated in accordance with Eq (3).

Electronic structure calculations were performed using the Gaussian 09 [5], and Molpro 2010 [6]. The gas phase enthalpies of formation of the ions comprising the salts **10a**, **10f**, and **10g** at $p^0$ = 1 bar and $T$ = 298.15 K ($\Delta_f H^0_{gas}$) were calculated using the explicitly correlated W1-F12 multi-level procedure [7] and the atomization energy approach described in detail elsewhere [5]. Note that the W1-F12 procedure employed in the present work had been slightly modified in comparison with the originally proposed technique: namely, the B3LYP-D3BJ/def2-TZVPP optimized geometries (the ZPE correction factor of 0.99) were used [8-9] and the diagonal Born-Oppenheimer corrections were omitted. The W1-F12 procedure employed in the present work was benchmarked on a series of energetic high-nitrogen heterocycles and polynitro compounds and exhibited a decent performance close to the average "thermochemical" accuracy (~4 kJ mol$^{-1}$) [10-13]. The multireference character of the wave functions of the species considered in the present work was estimated using the T1 diagnostic for the CCSD calculation [14]. The modest T1 values obtained in all cases (<0.020) justify the reliability of single reference-based electron correlation procedure employed in the present study. The heats of formation at 0 K for the elements in the gas phase $\Delta_f H^{0K}_{gas}$ (C) = 169.98 kcal mol$^{-1}$, $\Delta_f H^{0K}_{gas}$ (H) = 51.63 kcal mol$^{-1}$, $\Delta_f H^{0K}_{gas}$ (N) = 112.53 kcal mol$^{-1}$, and $\Delta_f H^{0K}_{gas}$ (O) = 58.99 kcal mol$^{-1}$ were taken from the NIST-JANAF tables [15].

**References:**


1. Kissinger, H.E. Reaction kinetics in differential thermal analysis. *Anal. Chem.* **1957**, *29*, (11), 1702–1706.
2. Jenkins, H.D.B.; Roobottom, H.K.; Passmore, J.; Glasser, L. Relationships among Ionic Lattice Energies, Molecular (Formula Unit) Volumes, and Thermochemical Radii. *Inorg. Chem.* **1999**, *38*, 3609–3620. https://doi.org/10.1021/ic9812961.
3. Jenkins, H.D.B.; Tudela, D.; Glasser, L. Lattice Potential Energy Estimation for Complex Ionic Salts from Density Measurements. *Inorg. Chem.* **2002**, *41*, 2364–2367. https://doi.org/10.1021/ic011216k.
4. Curtiss, L.A.; Raghavachari, K.; Redfern, P.C.; Pople, J.A. Assessment of Gaussian-2 and density functional theories for the computation of enthalpies of formation. *J. Chem. Phys.* **1997**, *106*, 1063. https://doi.org/10.1063/1.473182.

5. Frisch, M.J.; Trucks, G.W.; Schlegel, H.B.; Scuseria, G.E.; Robb, M.A.; Cheeseman, J.R.; Scalmani, G.; Barone, V.; Petersson, G.A.; Nakatsuji, H. et al. Gaussian 09, Revision D.01; Gaussian, Inc.: Wallingford, CT, 2016.
6. Werner, H.-J.; Knowles, P.J.; Knizia, G.; Manby, F.R.; Schutz, M.; Celani, P.; Korona, T.; Lindh, R.; Mitrushenkov, A.; Rauhut, G. et al. MOLPRO, version 2010.1, **2010**.
7. Karton, A.; Martin, J.M.L. Explicitly correlated Wn theory: W1-F12 and W2-F12 *J. Chem. Phys.* **2012**, *136*, 124114. https://doi.org/10.1063/1.3697678.
8. Kesharwani, M.K.; Brauer, B.; Martin, J.M.L. Frequency and Zero-Point Vibrational Energy Scale Factors for Double-Hybrid Density Functionals (and Other Selected Methods): Can Anharmonic Force Fields Be Avoided? *J. Phys. Chem. A* **2015**, *119*, 1701–1714. https://doi.org/10.1021/jp508422u.
9. Karton, A.; Schreiner, P.R.; Martin, J.M.L. Heats of formation of platonic hydrocarbon cages by means of high-level thermochemical procedures. *J. Comput. Chem.* **2016**, *37*, 49–58. https://doi.org/10.1002/jcc.23963.
10. Kiselev, V.G.; Goldsmith, C.F. Accurate Prediction of Bond Dissociation Energies and Barrier Heights for High-Energy Caged Nitro and Nitroamino Compounds Using a Coupled Cluster Theory. *J. Phys. Chem. A* **2019**, *123*, 4883–4890. https://doi.org/10.1021/acs.jpca.9b01506.
11. Kiselev, V.G.; Goldsmith, C.F. Accurate Thermochemistry of Novel Energetic Fused Tricyclic 1,2,3,4-Tetrazine Nitro Derivatives from Local Coupled Cluster Methods. *J. Phys. Chem. A* **2019**, *123*, 9818–9827. https://doi.org/10.1021/acs.jpca.9b08356.
12. Gorn, M.V.; Gritsan, N.P.; Goldsmith, C.F.; Kiselev, V.G. Thermal Stability of Bis-Tetrazole and Bis-Triazole Derivatives with Long Catenated Nitrogen Chains: Quantitative Insights from High-Level Quantum Chemical Calculations. *J. Phys. Chem. A* **2020**, *124*, 7665–7677. https://doi.org/10.1021/acs.jpca.0c04985.
13. Muravyev, N.V.; Monogarov, K.A.; Melnikov, I.N.; Pivkina, A.N.; Kiselev, V.G. Learning to Fly: Thermochemistry of Energetic Materials by Modified Thermogravimetric Analysis and Highly Accurate Quantum Chemical Calculations. *Phys. Chem. Chem. Phys.* **2021**, *23*, 15522–15542. https://doi.org/10.1039/d1cp02201f.
14. Lee, T.J.; Taylor, P.R. A diagnostic for determining the quality of single-reference electron correlation methods. *Int. J. Quantum Chem.* **1989**, *36*, 199–207. https://doi.org/10.1002/qua.560360824.
15. Chase, M.W., Jr. NIST-JANAF Thermochemical Tables. *J. Phys. Chem. Ref. Data*, 4th ed.; NIST-JANAF, **1998**; Mono. 9, Suppl. 1.

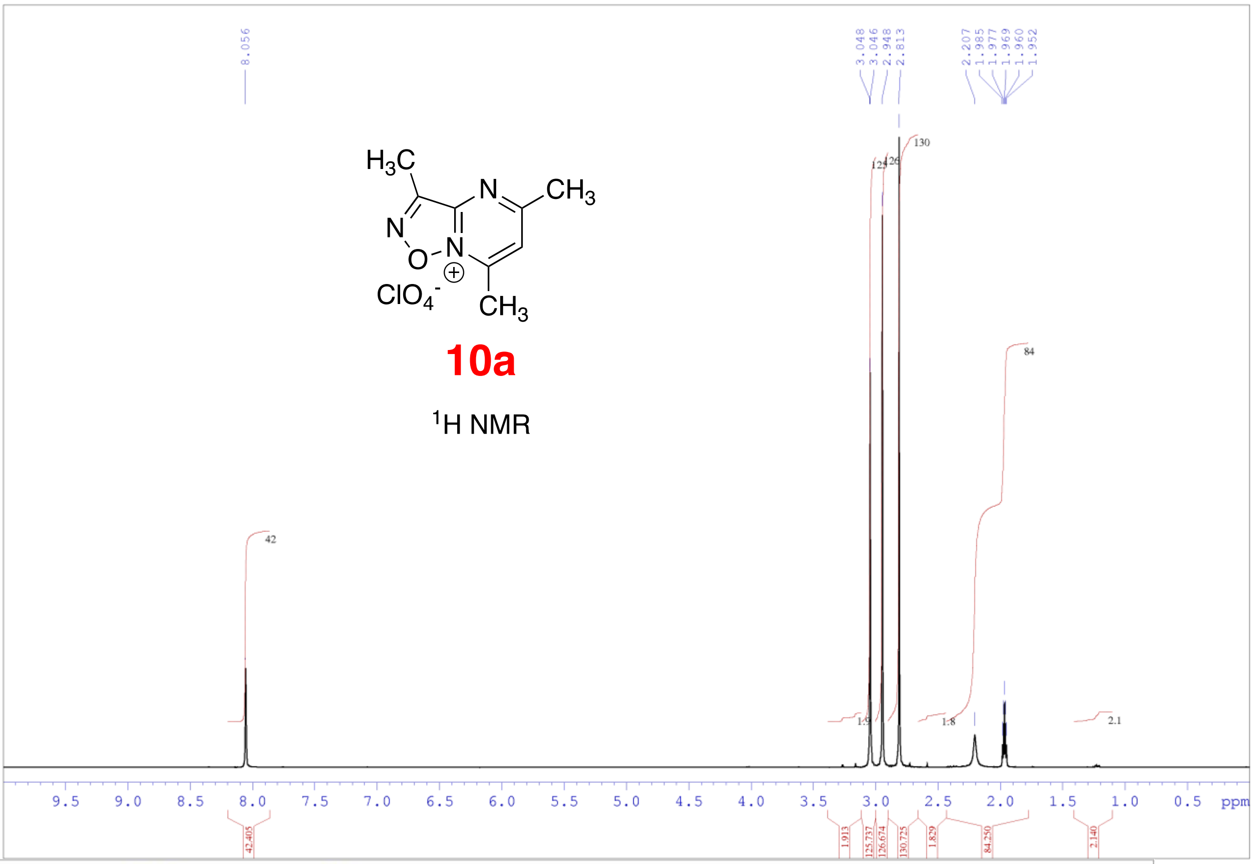
8.056
3.048
3.046
2.948
2.813
2.207
1.985
1.977
1.969
1.960
1.952
10a
1H NMR
9.5 9.0 8.5 8.0 7.5 7.0 6.5 6.0 5.5 5.0 4.5 4.0 3.5 3.0 2.5 2.0 1.5 1.0 0.5 ppm

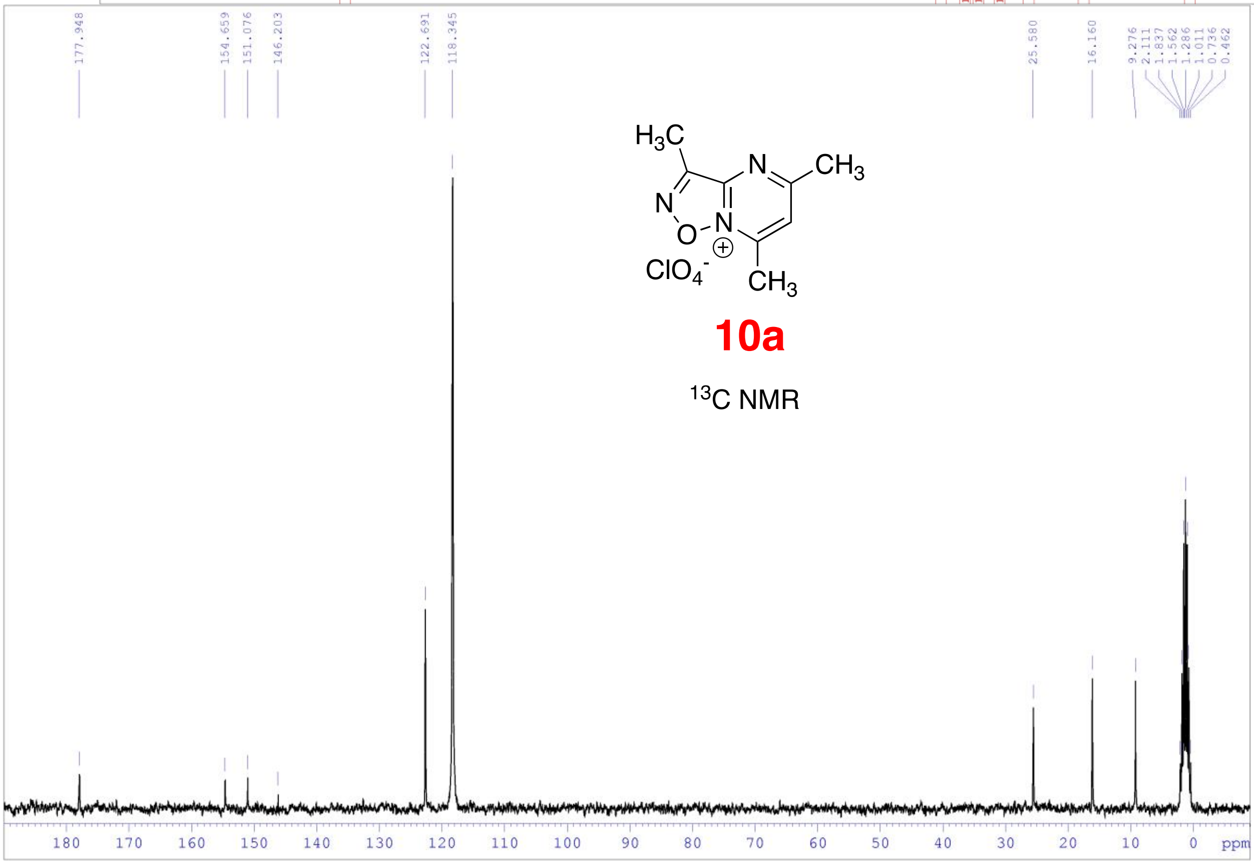
177.948
154.659
151.076
146.203
122.691
118.345
25.580
16.160
9.276
10a
13C NMR
180 170 160 150 140 130 120 110 100 90 80 70 60 50 40 30 20 10 0 ppm

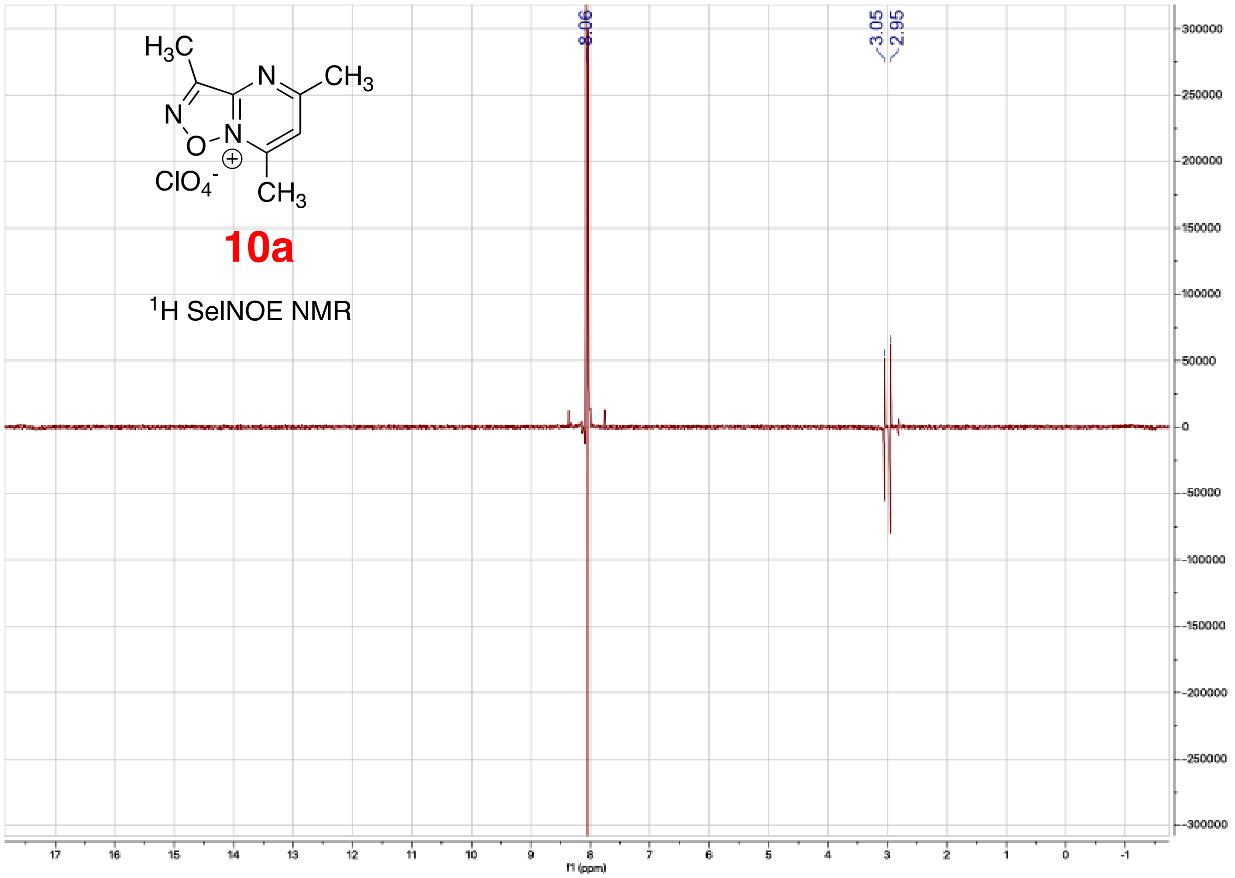
H3C
N
CH3
N
O
N
ClO4-
CH3
10a
1H SelNOE NMR
8.06
3.05
2.95
f1 (ppm)

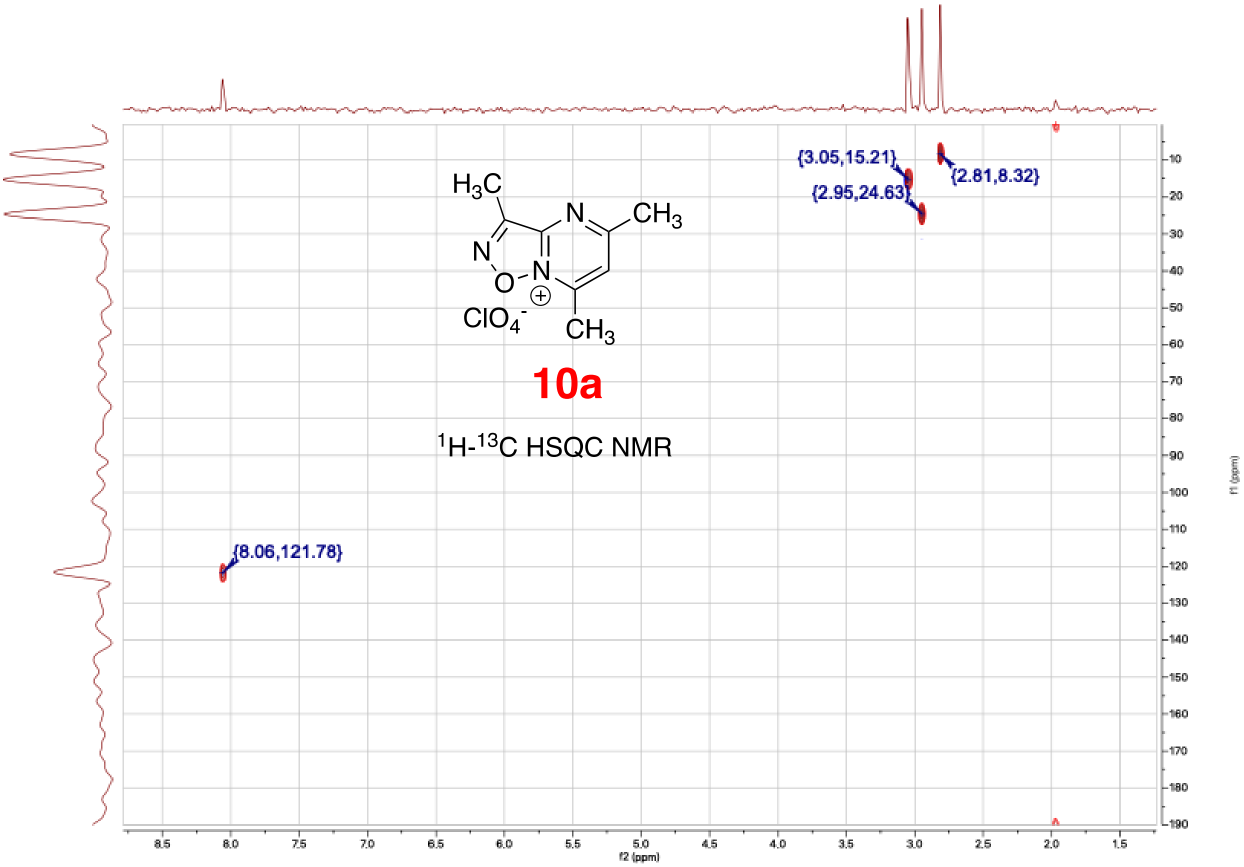
{3.05,15.21}
{2.81,8.32}
{2.95,24.63}
H3C
N
CH3
N
O
N
ClO4-
CH3
10a
1H-13C HSQC NMR
{8.06,121.78}
f2 (ppm)
f1 (ppm)

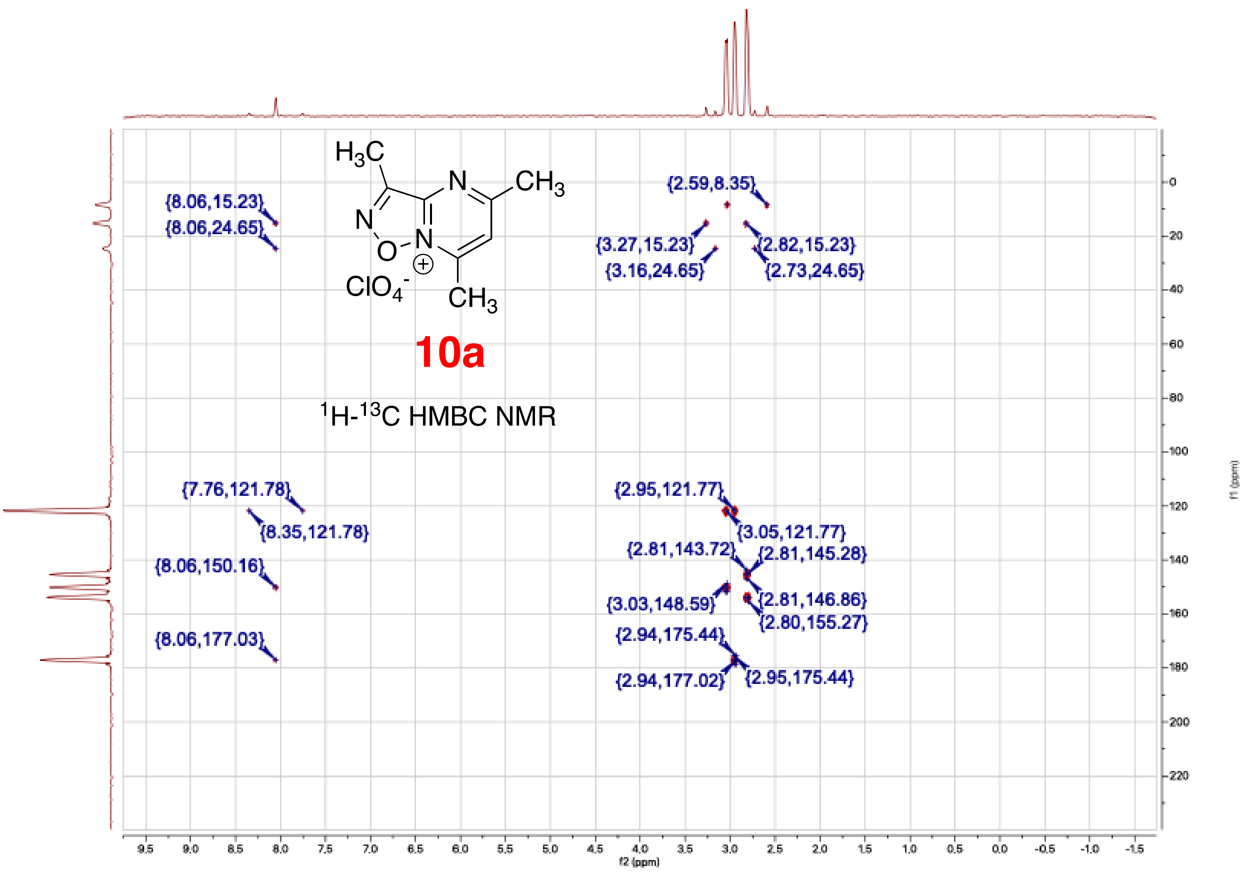

10a
$^1$H-$^{13}$C HMBC NMR
{8.06,15.23}
{8.06,24.65}
{2.59,8.35}
{3.27,15.23}
{2.82,15.23}
{3.16,24.65}
{2.73,24.65}
{7.76,121.78}
{8.35,121.78}
{8.06,150.16}
{8.06,177.03}
{2.95,121.77}
{3.05,121.77}
{2.81,143.72}
{2.81,145.28}
{3.03,148.59}
{2.81,146.86}
{2.80,155.27}
{2.94,175.44}
{2.94,177.02}
{2.95,175.44}
f2 (ppm)
f1 (ppm)


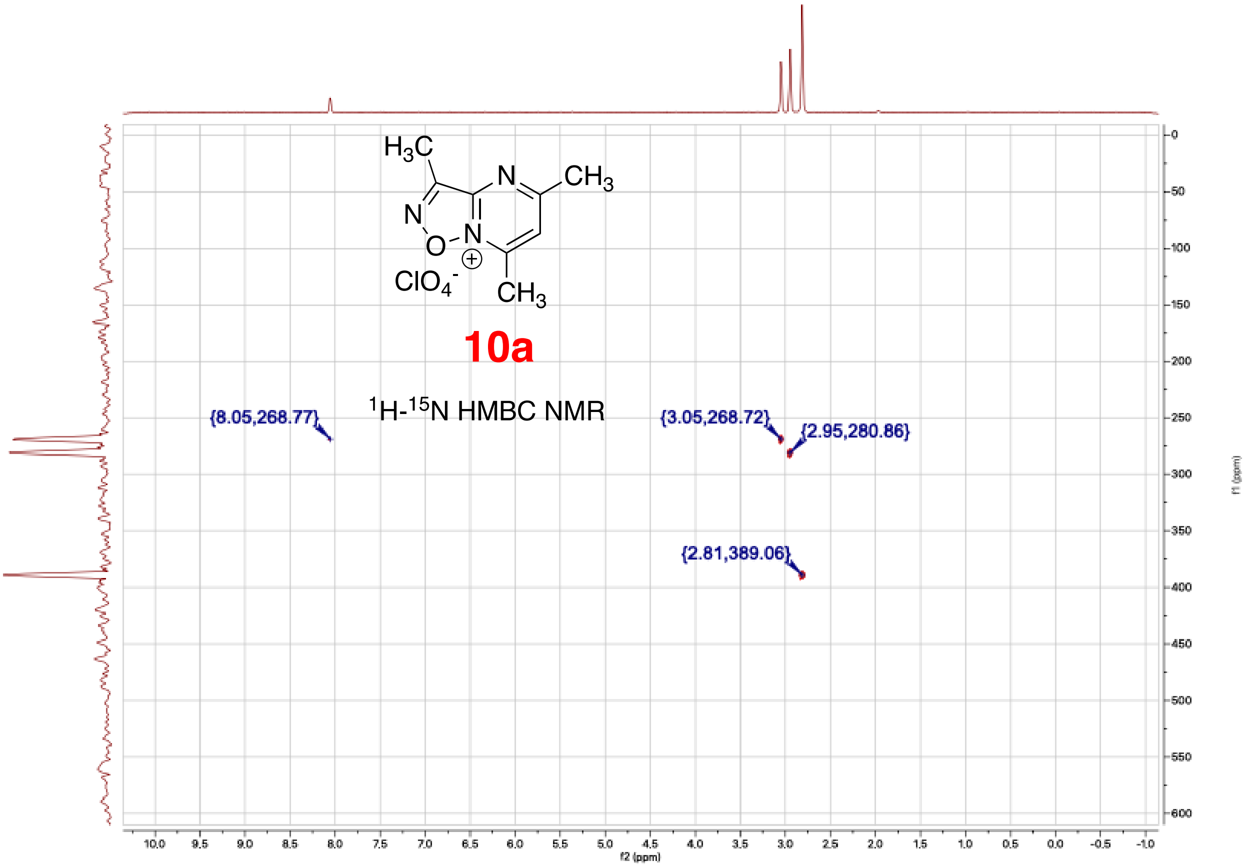

10a
$^1$H-$^{15}$N HMBC NMR
{8.05,268.77}
{3.05,268.72}
{2.95,280.86}
{2.81,389.06}
f2 (ppm)
f1 (ppm)

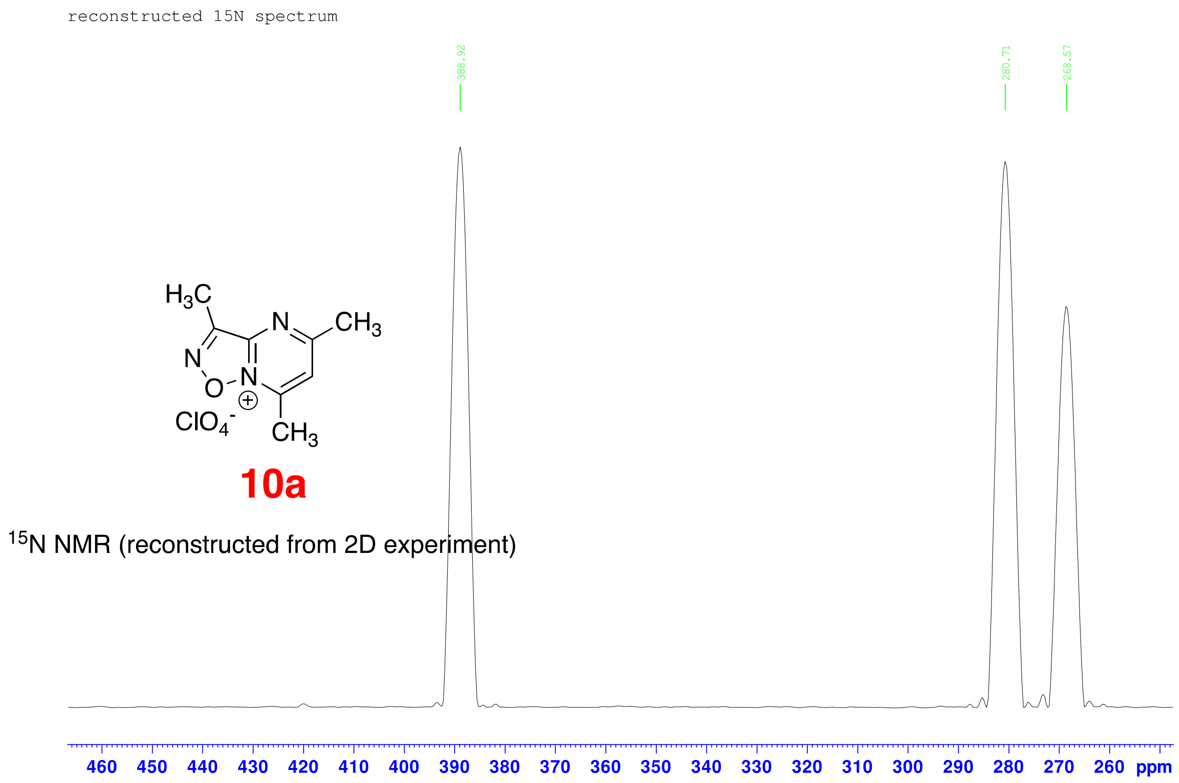
reconstructed 15N spectrum
388.92
280.71
268.57
H3C
N
CH3
N
O
N
ClO4-
CH3
10a
15N NMR (reconstructed from 2D experiment)
460 450 440 430 420 410 400 390 380 370 360 350 340 330 320 310 300 290 280 270 260 ppm

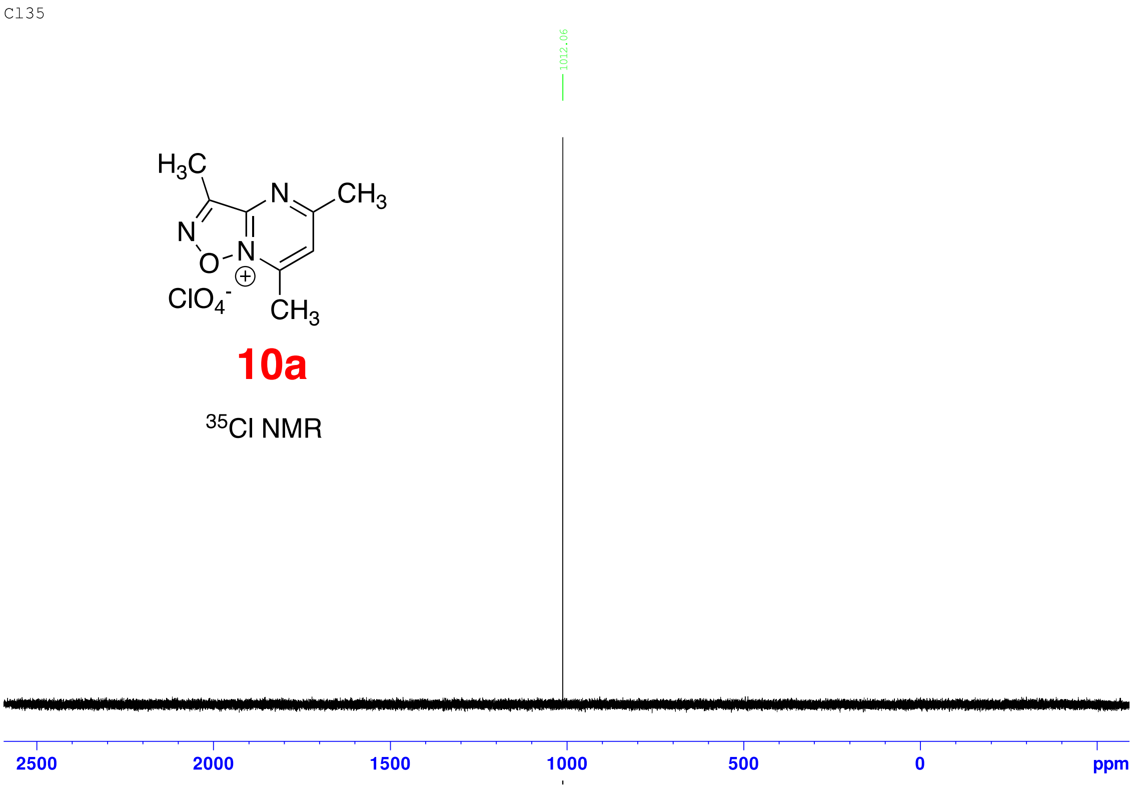
Cl35
1012.06
H3C
N
CH3
N
O
N
ClO4-
CH3
10a
35Cl NMR
2500 2000 1500 1000 500 0 ppm

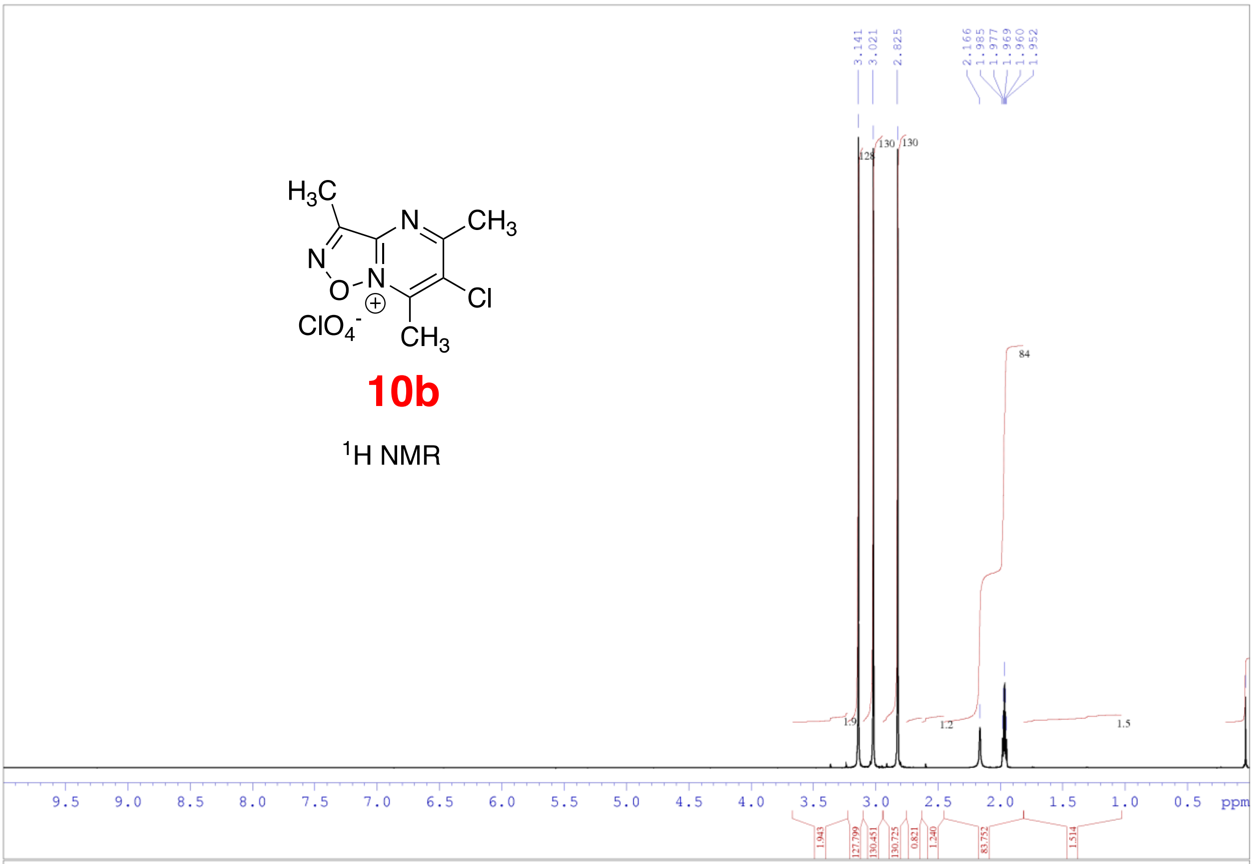
H3C
N
CH3
N
O
N
Cl
ClO4-
CH3
10b
1H NMR
3.141
3.021
2.825
2.166
1.985
1.977
1.969
1.960
1.952
9.5
9.0
8.5
8.0
7.5
7.0
6.5
6.0
5.5
5.0
4.5
4.0
3.5
3.0
2.5
2.0
1.5
1.0
0.5
ppm

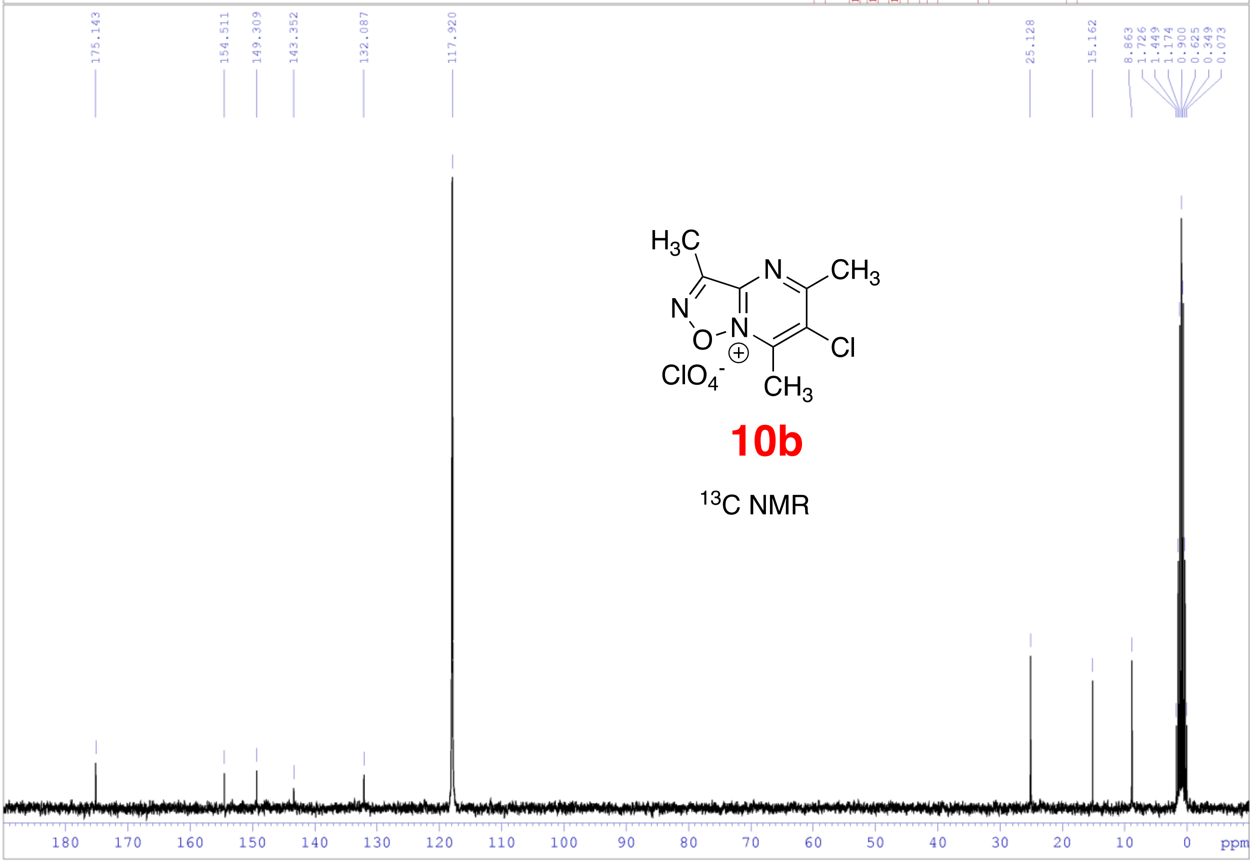
175.143
154.511
149.309
143.352
132.087
117.920
25.128
15.162
8.863
1.726
1.449
1.174
0.900
0.625
0.349
0.073
H3C
N
CH3
N
O
N
Cl
ClO4-
CH3
10b
13C NMR
180
170
160
150
140
130
120
110
100
90
80
70
60
50
40
30
20
10
0
ppm

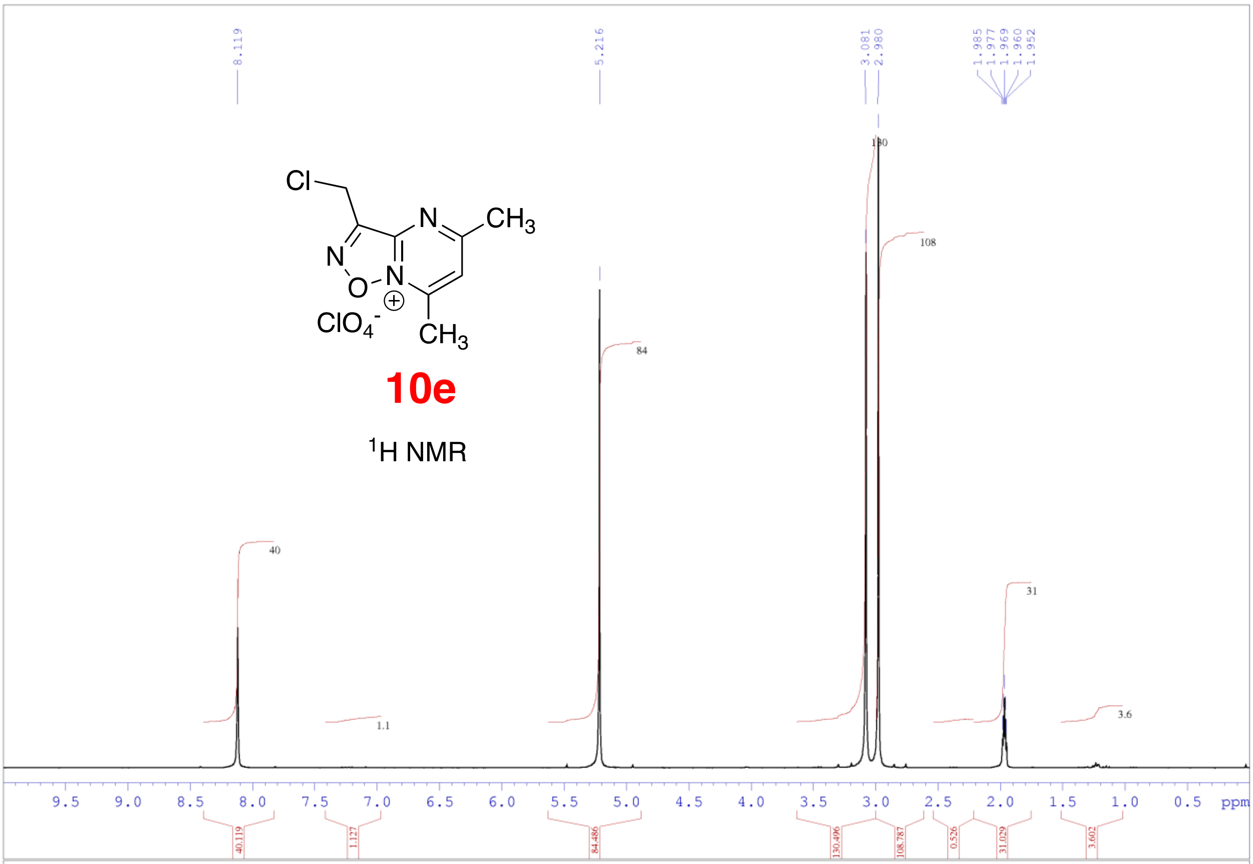
8.119
5.216
3.081
2.980
1.985
1.977
1.969
1.960
1.952
10e
$^1$H NMR
9.5 9.0 8.5 8.0 7.5 7.0 6.5 6.0 5.5 5.0 4.5 4.0 3.5 3.0 2.5 2.0 1.5 1.0 0.5 ppm
40.119
1.127
84.486
130.496
108.787
0.526
31.029
3.602

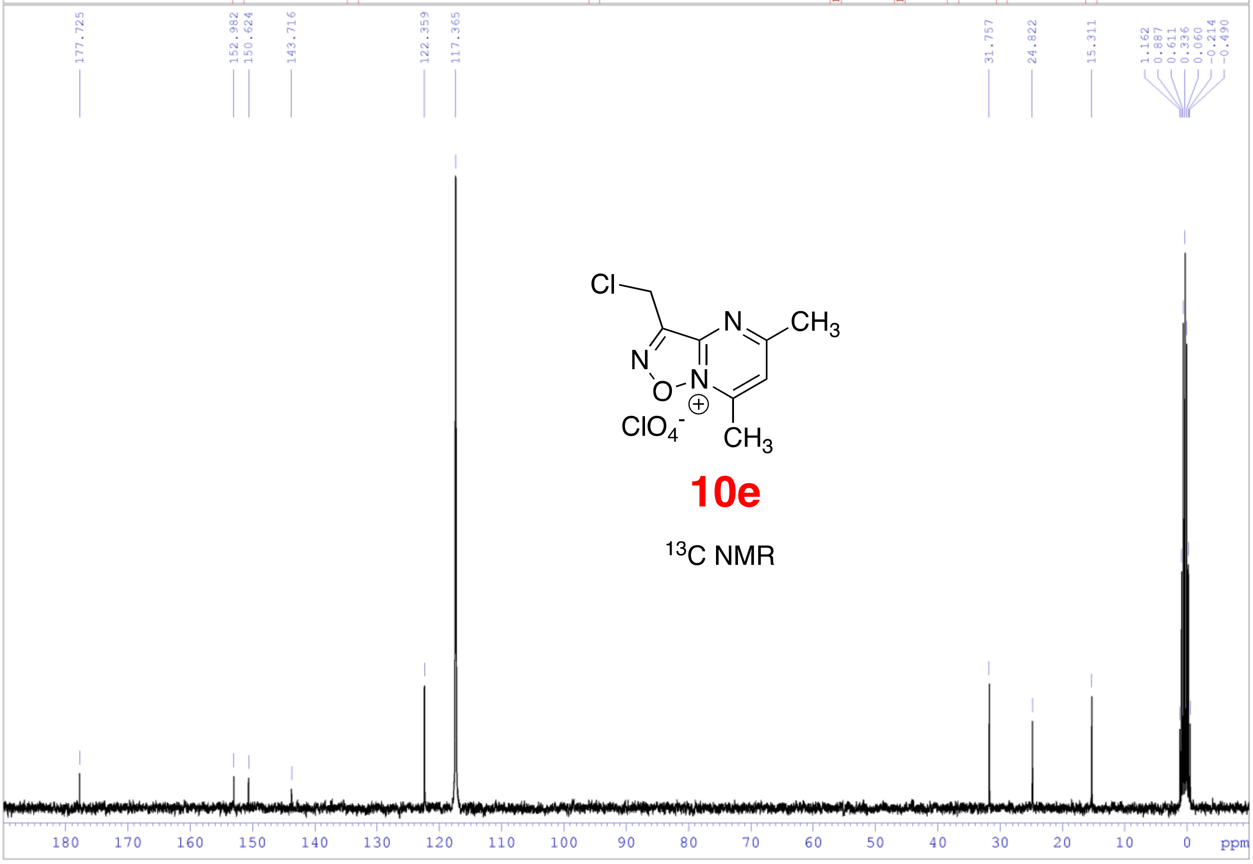
177.725
152.982
150.624
143.716
122.359
117.365
31.757
24.822
15.311
1.162
0.887
0.611
0.336
0.060
-0.214
-0.490
10e
$^{13}$C NMR
180 170 160 150 140 130 120 110 100 90 80 70 60 50 40 30 20 10 0 ppm

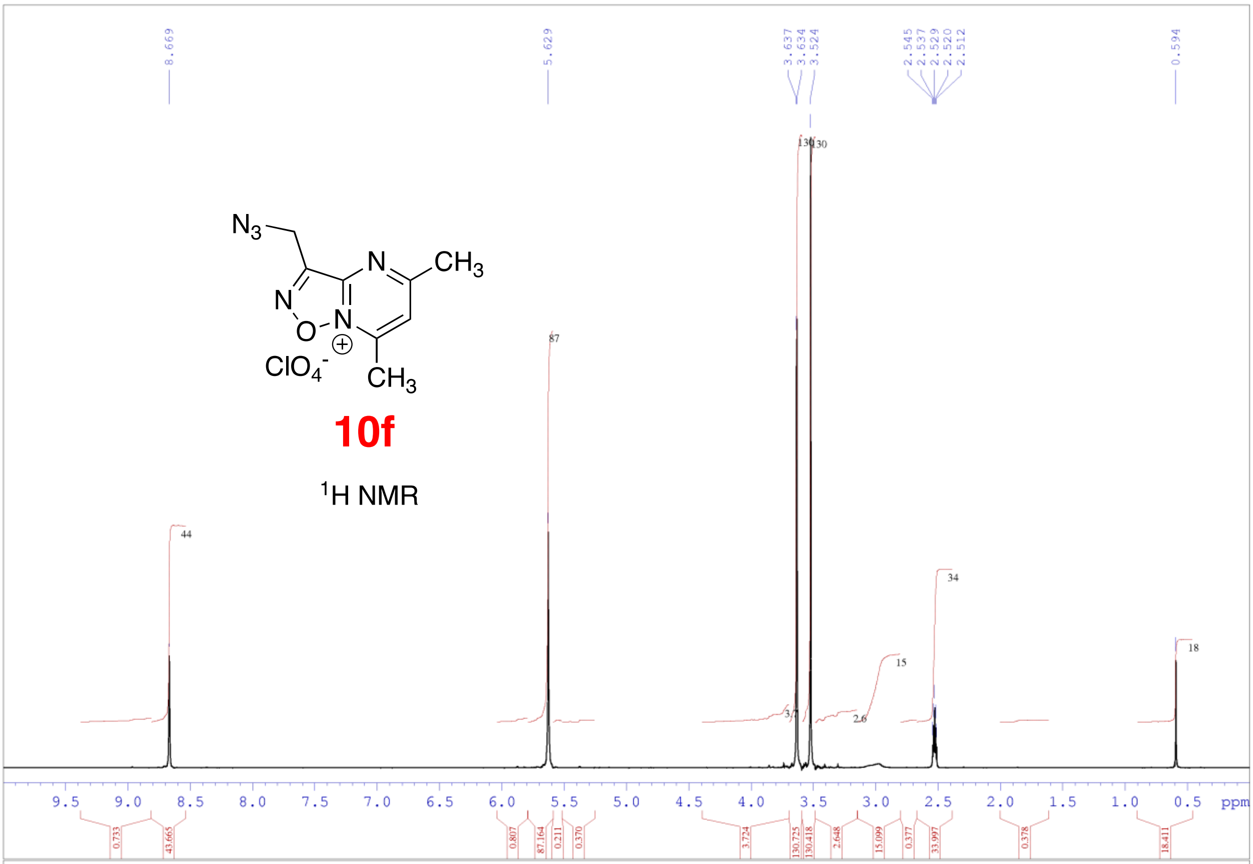

N3
N
CH3
N
O
N
ClO4-
CH3
10f
1H NMR
ppm


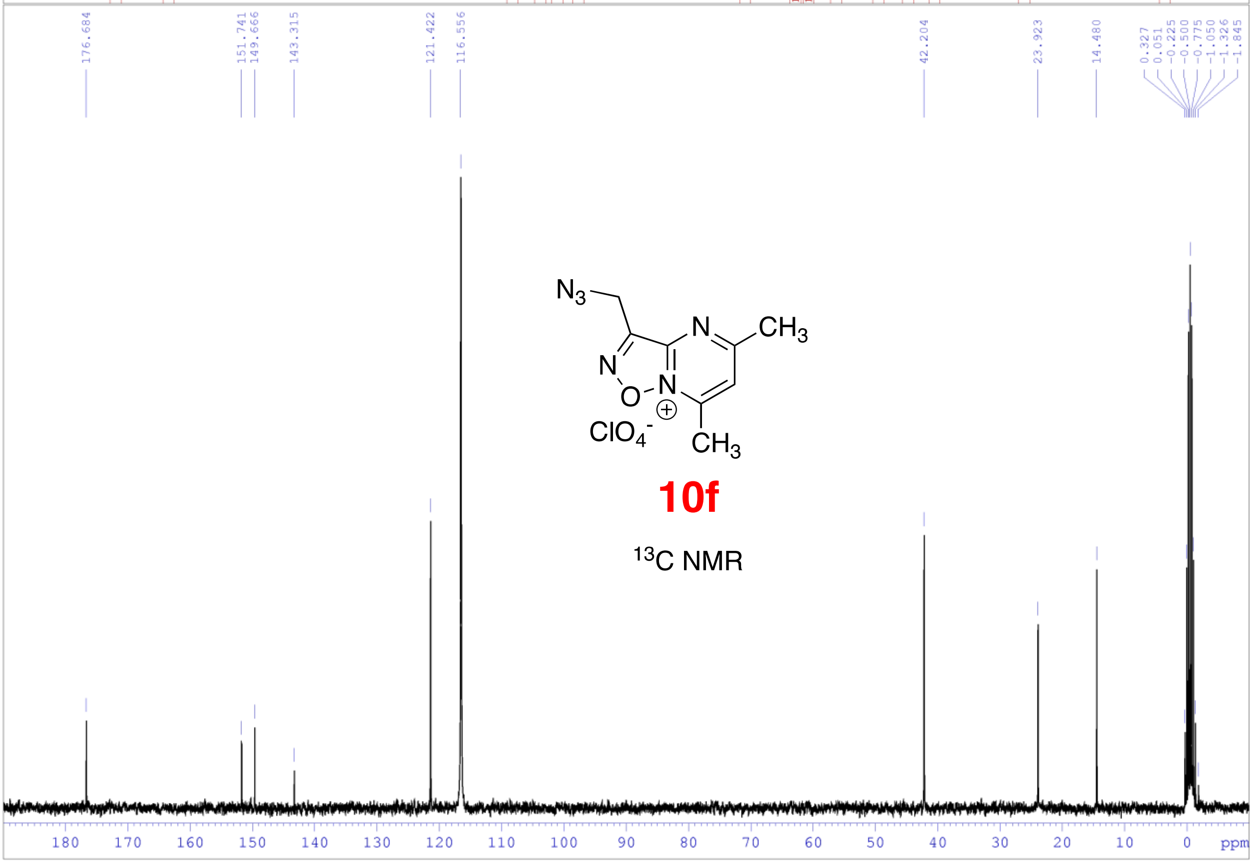

N3
N
CH3
N
O
N
ClO4-
CH3
10f
13C NMR
ppm

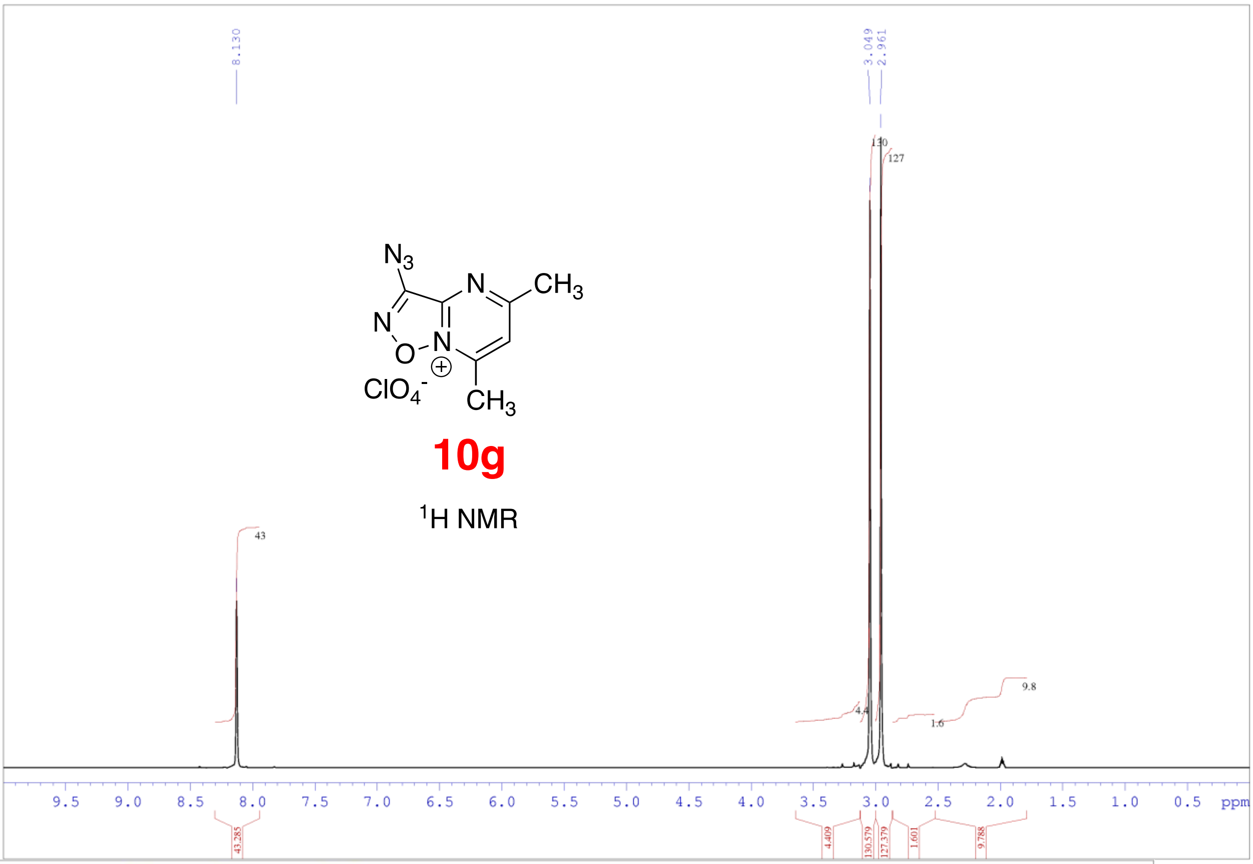
8.130
3.049
2.961
N3
N
CH3
N
O
N
ClO4-
CH3
10g
1H NMR
43
130
127
4.4
1.6
9.8
9.5
9.0
8.5
8.0
7.5
7.0
6.5
6.0
5.5
5.0
4.5
4.0
3.5
3.0
2.5
2.0
1.5
1.0
0.5
ppm
43.285
4.409
130.579
127.379
1.601
9.788

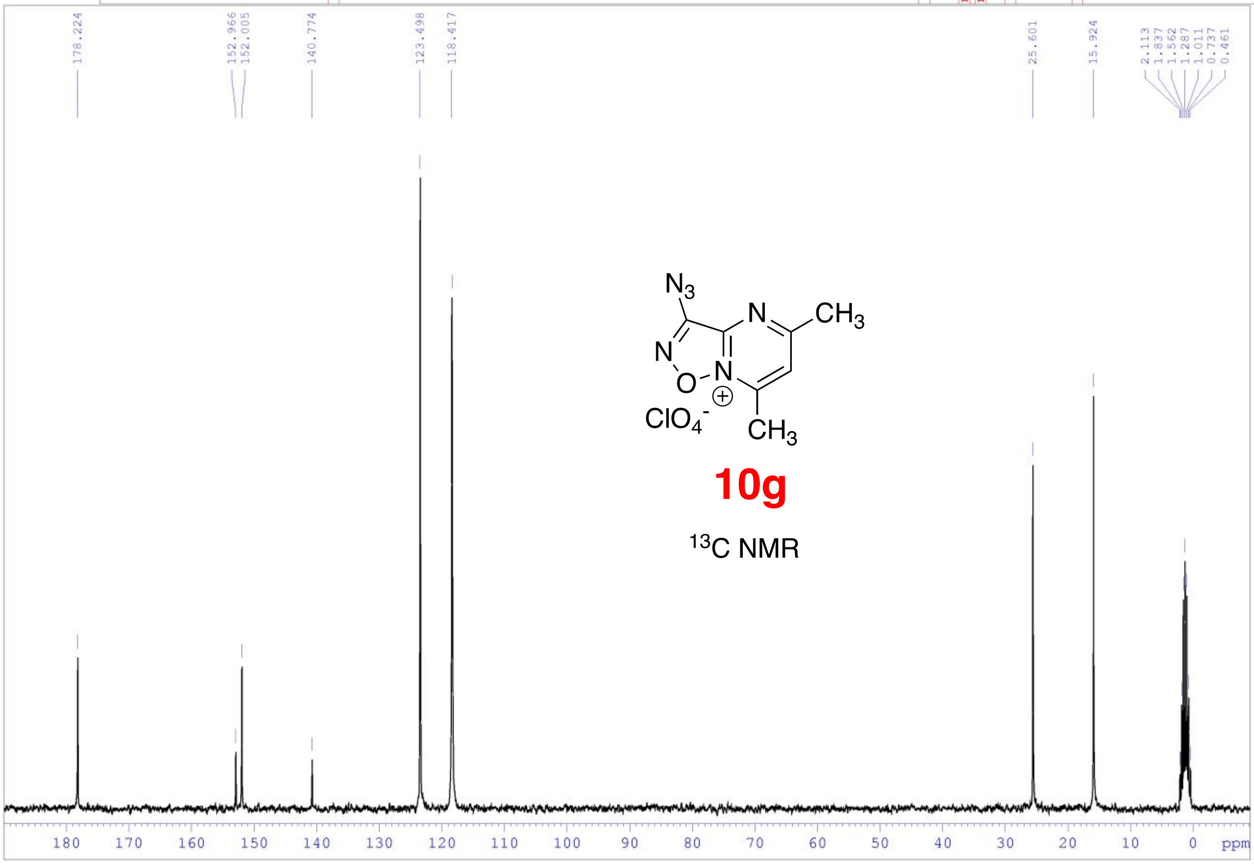
178.224
152.966
152.005
140.774
123.498
118.417
25.601
15.924
2.113
1.837
1.562
1.287
1.011
0.737
0.461
N3
N
CH3
N
O
N
ClO4-
CH3
10g
13C NMR
180
170
160
150
140
130
120
110
100
90
80
70
60
50
40
30
20
10
0
ppm

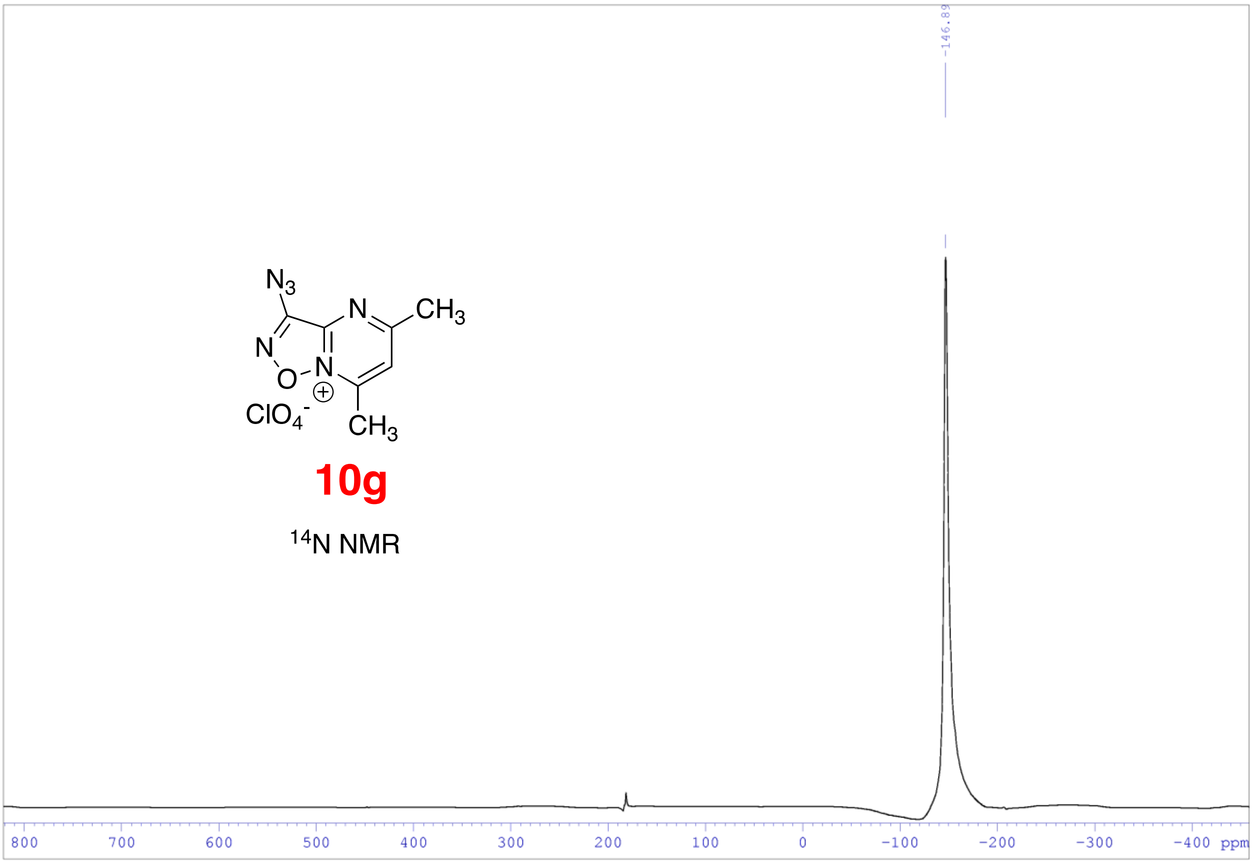
-146.89
N3
N
CH3
N
O
N
ClO4-
CH3
10g
14N NMR
800
700
600
500
400
300
200
100
0
-100
-200
-300
-400
ppm

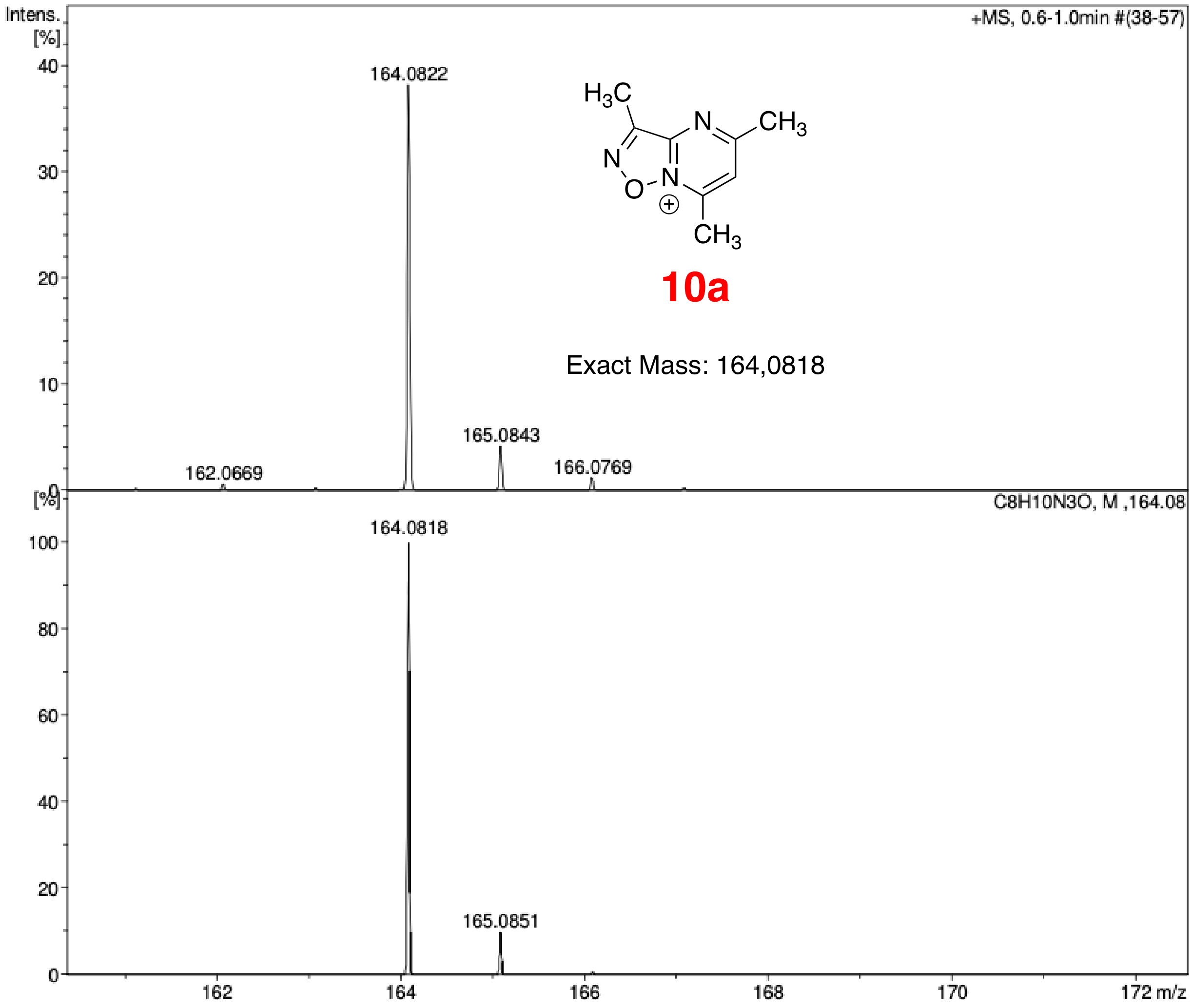
Intens. [%]
+MS, 0.6-1.0min #(38-57)
164.0822
162.0669
165.0843
166.0769
Exact Mass: 164,0818
C8H10N3O, M ,164.08
164.0818
165.0851
m/z

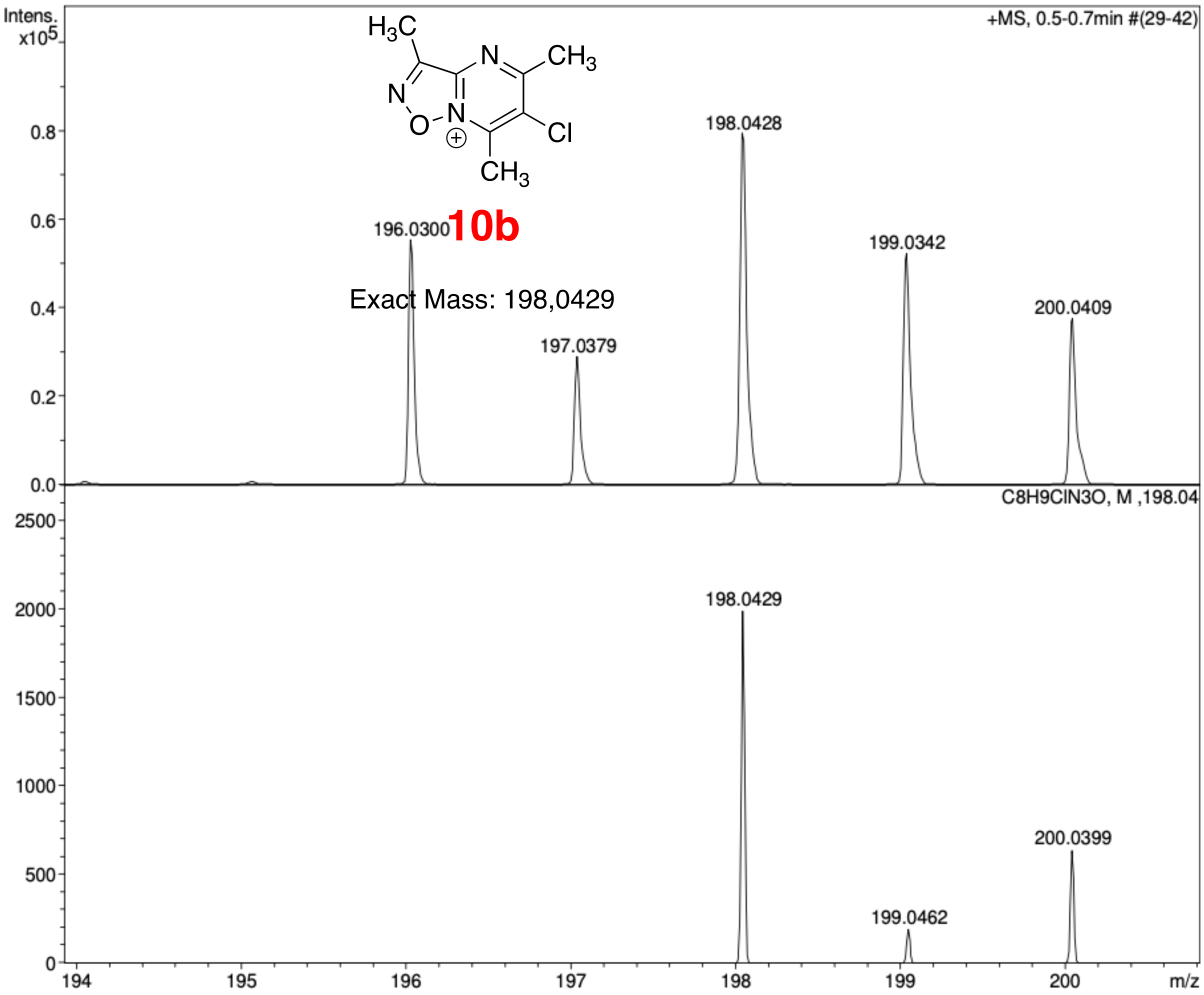
Intens.
x10^5
+MS, 0.5-0.7min #(29-42)
H3C
N
CH3
N
O
N
Cl
CH3
10b
Exact Mass: 198,0429
196.0300
197.0379
198.0428
199.0342
200.0409
C8H9ClN3O, M ,198.04
198.0429
199.0462
200.0399
0.0
0.2
0.4
0.6
0.8
0
500
1000
1500
2000
2500
194
195
196
197
198
199
200
m/z

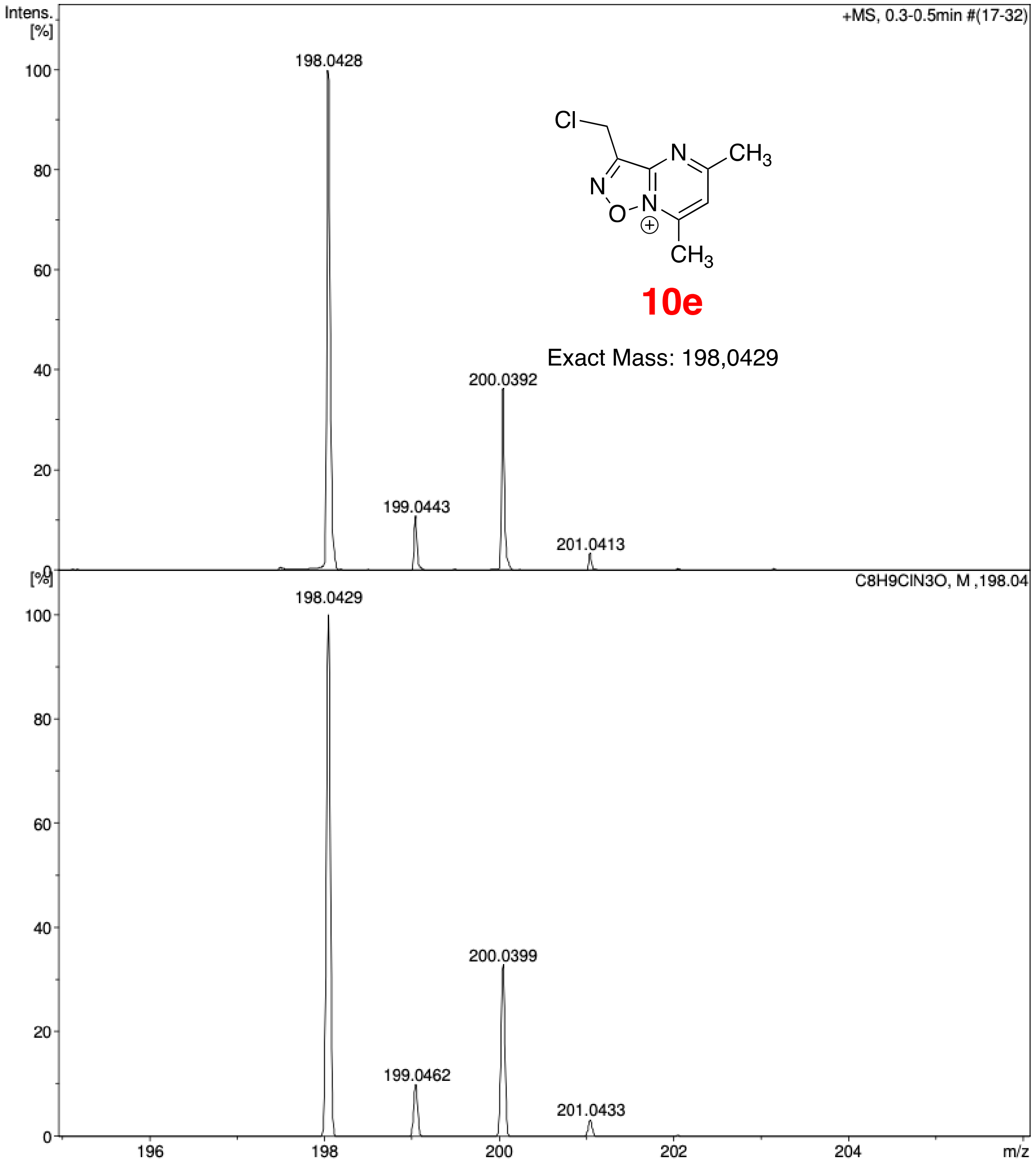

Intens. [%]
+MS, 0.3-0.5min #(17-32)
198.0428
199.0443
200.0392
201.0413
Cl
N
CH3
N
O
N
CH3
10e
Exact Mass: 198,0429
C8H9ClN3O, M ,198.04
198.0429
199.0462
200.0399
201.0433
m/z

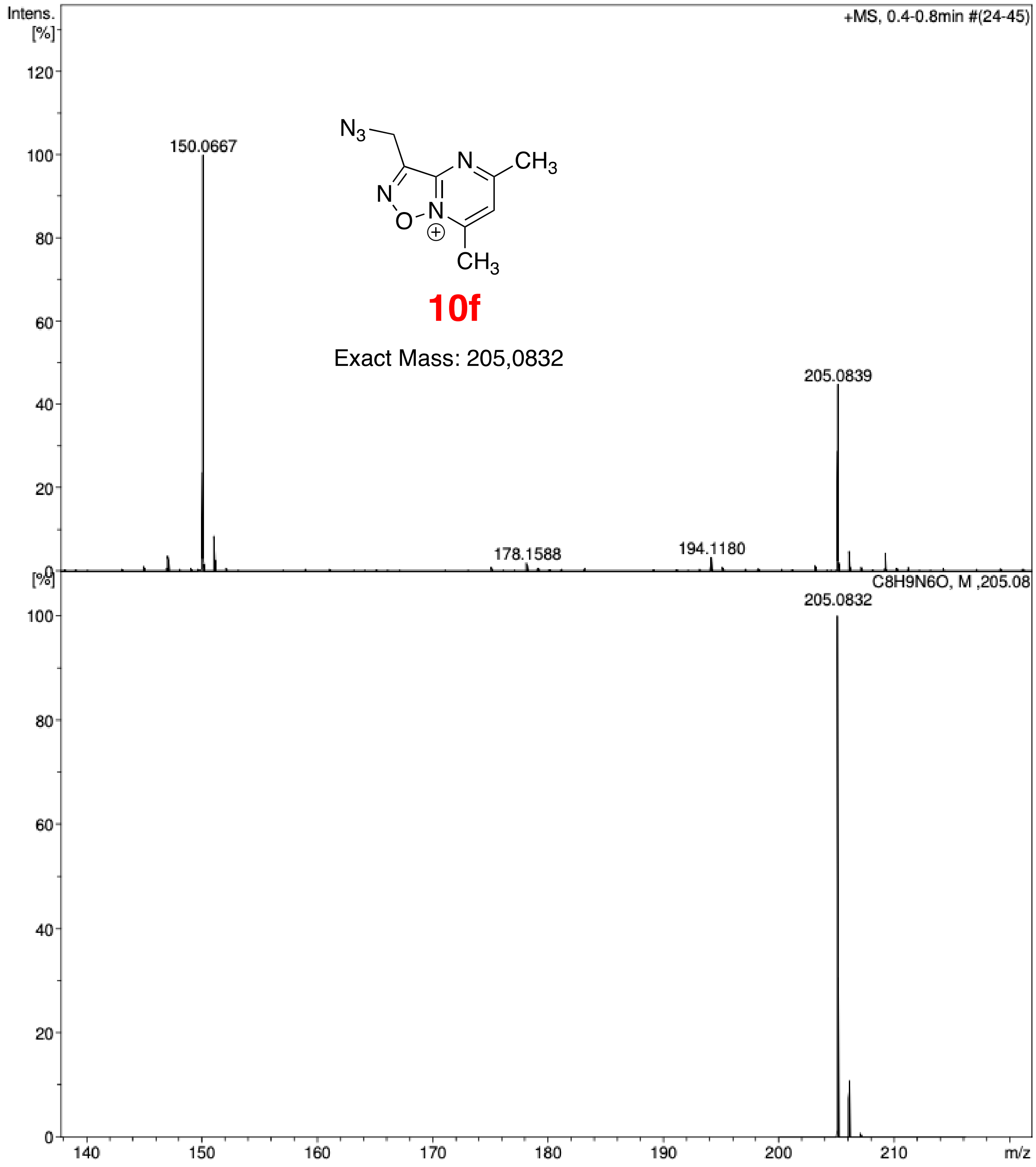
Intens.
[%]
+MS, 0.4-0.8min #(24-45)
150.0667
N3
N
CH3
N
O
N
CH3
10f
Exact Mass: 205,0832
205.0839
178.1588
194.1180
C8H9N6O, M ,205.08
205.0832
m/z

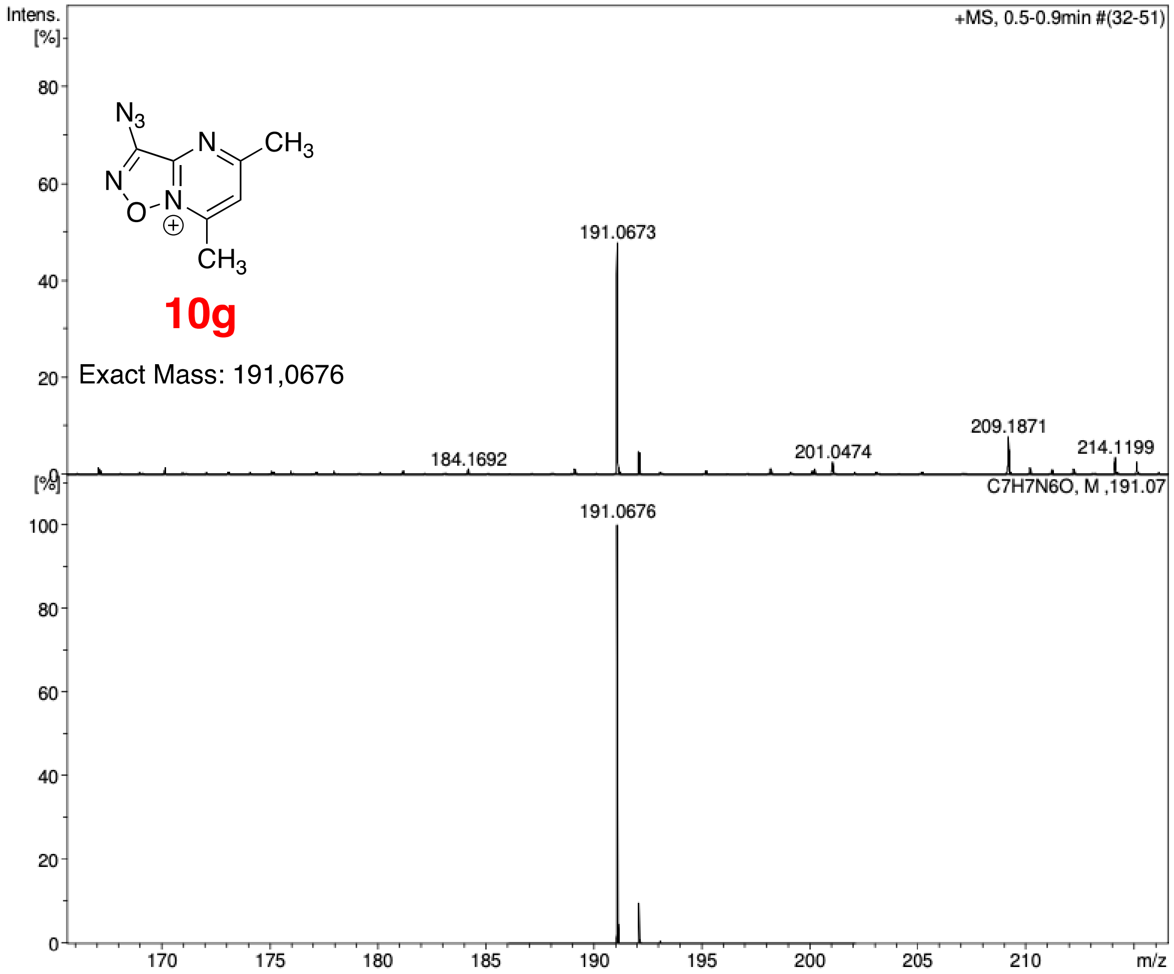
Intens.
[%]
+MS, 0.5-0.9min #(32-51)
N3
N
CH3
N
O
N
CH3
10g
Exact Mass: 191,0676
191.0673
184.1692
201.0474
209.1871
214.1199
C7H7N6O, M ,191.07
191.0676
170
175
180
185
190
195
200
205
210
m/z
